# Comparing genomic variant identification protocols for *Candida auris*

**DOI:** 10.1099/mgen.0.000979

**Published:** 2023-04-12

**Authors:** Xiao Li, José F. Muñoz, Lalitha Gade, Silvia Argimon, Marie-Elisabeth Bougnoux, Jolene R. Bowers, Nancy A. Chow, Isabel Cuesta, Rhys A. Farrer, Corinne Maufrais, Juan Monroy-Nieto, Dibyabhaba Pradhan, Jessie Uehling, Duong Vu, Corin A. Yeats, David M. Aanensen, Christophe d’Enfert, David M. Engelthaler, David W. Eyre, Matthew C. Fisher, Ferry Hagen, Wieland Meyer, Gagandeep Singh, Ana Alastruey-Izquierdo, Anastasia P. Litvintseva, Christina A. Cuomo

**Affiliations:** ^1^​ Broad Institute of MIT and Harvard, Cambridge, MA, 02142, USA; ^2^​ Mycotic Diseases Branch, Centers for Disease Control and Prevention, US Department of Health and Human Services, Atlanta, GA, 30329, USA; ^3^​ Centre for Genomic Pathogen Surveillance, Big Data Institute, University of Oxford, Oxford, UK; ^4^​ Institut Pasteur, Université Paris Cité, INRAE, USC2019, Unité Biologie et Pathogénicité Fongiques, Paris, France; ^5^​ Université Paris Cité, Hôpital Necker-Enfants-Malades, Unité de Parasitologie-Mycologie, Assistance Publique des Hôpitaux de Paris, Paris, France; ^6^​ Translational Genomics Research Institute, Pathogen and Microbiome Division, Flagstaff, AZ 86005, USA; ^7^​ Mycology Reference Laboratory, National Centre for Microbiology, Instituto de Salud Carlos III, Madrid, Spain; ^8^​ Medical Research Council Centre for Medical Mycology, University of Exeter, Exeter, EX4 4PY, UK; ^9^​ Institut Pasteur, Université Paris Cité, CNRS USR 3756, Hub de Bioinformatique et Biostatistique, Paris, France; ^10^​ All India Institute of Medical Sciences, Ansari Nagar, New Delhi, 110029, India; ^11^​ Botany and Plant Pathology, Oregon State University, Corvallis, OR 97330, USA; ^12^​ Westerdijk Fungal Biodiversity Institute, Uppsalalaan 8, 3584CT, Utrecht, Netherlands; ^13^​ NIHR Oxford Biomedical Research Centre, University of Oxford, Oxford, UK; ^14^​ MRC Centre for Global Infectious Disease Analysis, Imperial College London, London, UK; ^15^​ Institute for Biodiversity and Ecosystem Dynamics (IBED), University of Amsterdam, Amsterdam, Netherlands; ^16^​ Department of Medical Microbiology, University Medical Center Utrecht, Utrecht, Netherlands; ^17^​ Sydney Medical School, University of Sydney, Sydney, NSW 2050, Australia

**Keywords:** fungal genomics, whole-genome sequencing, variant calling pipelines, benchmarking, Candida

## Abstract

Genomic analyses are widely applied to epidemiological, population genetic and experimental studies of pathogenic fungi. A wide range of methods are employed to carry out these analyses, typically without including controls that gauge the accuracy of variant prediction. The importance of tracking outbreaks at a global scale has raised the urgency of establishing high-accuracy pipelines that generate consistent results between research groups. To evaluate currently employed methods for whole-genome variant detection and elaborate best practices for fungal pathogens, we compared how 14 independent variant calling pipelines performed across 35 *Candida auris* isolates from 4 distinct clades and evaluated the performance of variant calling, single-nucleotide polymorphism (SNP) counts and phylogenetic inference results. Although these pipelines used different variant callers and filtering criteria, we found high overall agreement of SNPs from each pipeline. This concordance correlated with site quality, as SNPs discovered by a few pipelines tended to show lower mapping quality scores and depth of coverage than those recovered by all pipelines. We observed that the major differences between pipelines were due to variation in read trimming strategies, SNP calling methods and parameters, and downstream filtration criteria. We calculated specificity and sensitivity for each pipeline by aligning three isolates with chromosomal level assemblies and found that the GATK-based pipelines were well balanced between these metrics. Selection of trimming methods had a greater impact on SAMtools-based pipelines than those using GATK. Phylogenetic trees inferred by each pipeline showed high consistency at the clade level, but there was more variability between isolates from a single outbreak, with pipelines that used more stringent cutoffs having lower resolution. This project generated two truth datasets useful for routine benchmarking of *C. auris* variant calling, a consensus VCF of genotypes discovered by 10 or more pipelines across these 35 diverse isolates and variants for 2 samples identified from whole-genome alignments. This study provides a foundation for evaluating SNP calling pipelines and developing best practices for future fungal genomic studies.

## Data Summary

All Illumina sequences generated by this project are available in the National Center for Biotechnology Information (NCBI) Sequence Read Archive (SRA) under BioProjects PRJNA328792, PRJNA470683, PRJNA493622 and PRJNA595978 (Table S1, available with the online version of this article). Submitted data for each participating group (raw VCF, pairwise SNP matrix and FASTA alignment), the standardized versions of these files used for comparison, and the variants for CA05 and CA06 using whole-genome alignment with B8441 are available in FigShare and are linked to the GitHub repository containing all code used to process and compare these data (https://figshare.com/projects/Genomic_epidemiology_of_Candida_auris_-_Benchmarking_Variants_Identification/86372).

**Impact Statement**

The widespread use of genomic data in various epidemiological and experimental studies underscores the importance of generating consistent data that are comparable between different groups. To address the need for standardization between different pipelines used for genomic analyses, we chose the genome of *Candida auris* as a case study due the urgency of tracking this emerging fungal pathogen and to harmonize ongoing genomic efforts undertaken by several groups studying its unprecedented emergence. To compare the data outputs of different pipelines, we provided Illumina sequence reads for 35 isolates representing each of the 4 major clades, encompassing some isolates from the same outbreak in the New York City area [1]. Our analysis found a high degree of concordance between pipelines, including the identification of single-nucleotide polymorphisms (SNPs) associated with drug resistance; however, there was a higher variability in inferring the relationship between isolates from a single outbreak. By comparing different methods and developing truth datasets, this study provides a framework for further standardization and evaluation of SNP calling methods to ensure that results are comparable between independent studies.

## Introduction

Genomic analyses are used in a wide variety of studies to understand the evolutionary history, population structure, mechanisms of virulence and drug resistance in fungi. This approach has been applied to trace the global emergence of several novel human, animal and plant pathogens, including *Candida auris*, *Batrachochytrium dendrobatidis*, *Magnaporthe oryzae* and other pathogens of clinical or economic significance [2–7]. Genomic analyses rely on identification of genomic variants, most often single-nucleotide polymorphisms (SNPs), which are then used to estimate genetic relationships among isolates. The variant calling process is subject to potential confounding factors at each step, and there is often considerable variation between results produced by different pipelines. Accurate prediction of variation between genomes and standardization between methods are especially important in outbreak investigations, where molecular genotyping data are used to make public health decisions about tracing and containing the spread of infection.

The potential sources of variability between pipelines are associated with nearly every step, including the choice of reference genomes, sequencing platform, preprocessing of reads, alignment and variant calling methods, and SNP filtering criteria. Many studies use a resequencing approach that compares short reads to a reference genome [8]. The quality of the reference assembly, in terms of both continuity and accuracy, and the selection of a reference that is representative of the population under study, are essential for the accurate identification of whole-genome variants [9, 10]. Selection of a phylogenetically distant reference may result in lower power to detect variants, particularly in highly divergent or unique regions. Methods used for aligning reads to a reference as well as genome-specific properties, such as the frequency, size and identity of repetitive or low-complexity regions, regions with extreme GC content, and the presence of regions unique to a set of genomes, impact on the quality and genome-wide coverage of read alignments. In addition, read length and quality may impact on mapping accuracy, especially in repetitive regions. Finally, the selection of variant calling methods and parameters can also make a significant difference to the quality of the final variant set. Among the methods that identify variants from read alignments, GATK [11] and SAMtools [12] are frequently used in combination with post-calling filtering parameters that tend to vary between different organisms and studies. The choices made at each of these steps affect the sensitivity and precision of the call set [13].

While best practices have been recommended for some variant calling workflows, these methods have not been rigorously benchmarked and validated for fungal genomes [14]. In part, this is due to the lack of truth sets of validated variants between isolates. Here, we compare 14 SNP calling methods for *C. auris* utilized by 11 independent research groups. Each group used a dataset of 35 isolates representing each of the 4 major *C. auris* clades [5], including closely related isolates from the same outbreak. This species was selected for this comparison due to its importance as an emerging human pathogenic fungus causing worldwide clinical outbreaks of multidrug-resistant infections [3]. In addition, multiple groups have published genomic analyses of *C. auris* outbreaks using SNPs called by different pipelines [5, 15–17], highlighting the need to understand differences between these methods. While an initial genomic analysis used results from two different pipelines to confirm the unusual population structure of this species [5], differences between pipelines have not been studied in detail. Our results indicate that, while variation in the processing steps resulted in variation in the total number of variants, the vast majority of sites were called at high fidelity by all pipelines. We benchmarked the overall accuracy of each method using a truth set identified by comparing chromosome level genome assemblies. Our results highlight factors to consider in selecting a method, data processing and SNP filtering criteria, and provide resource datasets for future benchmarking.

## Methods

### Whole-genome sequencing (WGS) of *C. auris* isolates

Illumina WGS for 35 *C*. *auris* isolates representing each of the 4 clades (I, II, III and IV, initially isolated from South Asia, East Asia, Africa and South America, respectively) [5] was provided for analysis (Table S1, available with the online version of this article). DNA was extracted using the ZR Fungal/Bacterial DNA MiniPrep kit (Zymo Research, Irvine, CA, USA). Genomic libraries were constructed and barcoded using the NEBNext Ultra DNA Library Prep kit for Illumina (New England Biolabs, Ipswich, MA, USA). Libraries were sequenced on either the Illumina HiSeq 2500 platform (Illumina, San Diego, CA, USA) using the HiSeq Rapid SBS kit v2 500-cycles in the rapid mode or the MiSeq platform using the MiSeq Reagent kit v2 500-cycles to generate paired 250 base reads with average depth of coverage ranging from 40–230× (mean 137×; Table S1).

### Variant calling methods for 14 pipelines

An invitation to participate in the study was sent to all members of the ISHAM Working Group on Genomic Epidemiology of Fungal Pathogens (https://www.isham.org/working-groups/genomic-epidemiology-fungal-infections). All that agreed to participate received an email with instructions to participate and to download the dataset of Illumina raw sequences for 35 isolates. Many of the samples contained more than one FASTQ read file per isolate, which most groups merged by sample before processing. All groups were requested to use the same reference genome for isolate B8441 (https://www.ncbi.nlm.nih.gov/nuccore/PEKT00000000.2/) [5, 18] and to provide detailed methods, a standard VCF file, a pairwise matrix of SNPs counts and a maximum parsimony phylogenetic tree inferred using the predicted variants. In total, 14 datasets were submitted by 11 different groups; 3 groups submitted results from 2 different pipelines. Detailed descriptions of the pipeline used to generate each dataset are provided below.

### Pipeline 01 – CFSAN

The pipeline WGS-Outbreaker (https://github.com/BU-ISCIII/WGS-Outbreaker) was used for SNP identification. Read quality was assessed with FastQC v0.11.8 (https://www.bioinformatics.babraham.ac.uk/projects/fastqc/) and reads were trimmed with Trimmomatic v0.32 with parameters ILLUMINACLIP:/opt/Trimmomatic-0.33/adapters/TruSeq3-PE.fa:2 : 30 : 10 TRAILING:10 SLIDINGWINDOW:4 : 15 MINLEN:70 [19] and aligned with BWA MEM v0.7.12. SNPs were called and an SNP matrix was constructed using the CFSAN SNP-pipeline v2.0 (https://github.com/CFSAN-Biostatistics/snp-pipeline) with filtering criteria: minBaseQual: 0 (disable base quality filtering), minConsFreq: 0.6 (minimum 0.6 allele frequency for calling a variant in the consensus step), minConsStrdDpth: 0 (disabled strand bias) and minConsStrdBias: 0 (disabled strand bias in the consensus step determination). A phylogenetic tree was constructed using maximum likelihood using RAxML v8.2.9 (inference parameters: ‘-m GTRCAT -V -w’ with 100 bootstrap duplicates).

### Pipeline 02 – GATK v3.6

This pipeline is an adaptation of the one used to analyse the diploid genome of *Candida albicans* [4]. It was optimized for *C. albicans*, and for this analysis a few adaptions were made. Read quality was assessed with FastQC v0.11.5. Reads were trimmed with cutadapt [20] with ‘-q 30 -e 0.1 n 3 -O 6 m 30’ options and aligned with BWA MEM v0.7.1 [21]. SAMtools v1.9 [12] and Picard tools v1.94 (http://broadinstitute.github.io/picard) were then used to filter, sort and convert SAM files. Variants were called with GenomeAnalysisTK v3.6 HaplotypeCaller with ploidy=1, stand_call_conf=30.0 and stand_emit_conf=10.0 options [22]. SNPs were selected with GATK SelectVariants and filtered with GATK VariantFiltration [FisherStrand (FS) >60.0, RMSMappingQuality (MQ) <40.0, QualByDepth (QD) <2.0, Coverage (DP) <10] [11]. Variants were combined with GATK CombineVariants (--filteredrecordsmergetype KEEP_IF_ANY_UNFILTERED). For each isolate, specific filtrations were performed with a custom Python v2.7 script (https://github.com/maufrais/Scripts/blob/master/SNPs/variant_filtarion.py): individual variants were removed if the depth of coverage (DP) <10, the genotype quality (GQ) <80 and the homozygous allele balance (ABHom) <0.9. A dataset of 228 820 sites was created where filtered-out variants were replaced by a gap. The phylogenetic tree was generated using Phylip v3.67 Maximum Parsimony dnapars tool [23], where the method of Fitch is used to count the number of changes of base needed on a given tree.

### Pipeline 03 – GATK v3.7.9

FASTQ files were converted to unaligned BAMs using Picard v1.782 (http://broadinstitute.github.io/picard /). The library origin was conserved for those samples that have more than one set of paired-end reads. Then, samples were processed using a pipeline implemented in the workflow description language (WDL) to run on a local compute cluster via Cromwell (https://github.com/broadinstitute/cromwell). In this pipeline, reads were aligned to the *C. auris* assembly of isolate B8441 (PEKT00000000.2) using BWA-MEM v0.7.12 [21]. BAM files were processed using SAMtools (sort), Picard (MarkDuplicates), and SAMtools (reorder) [12]. Regions with indels were locally realigned (GATK v3.7.93 RealignerTargetCreator and IndelRealigner) [11]. Variants were then identified using GATK HaplotypeCaller in GVCF mode with ploidy=1 and gVCFs combined with CombineGVCFs, and then genotypeGVCFs was used to predict variants in each strain [22]. A combined VCF generated by GatherVCFs was filtered using GATK’s VariantFiltration with QD <2.0, FS >60.0 and MQ <40.0. Individual genotypes were then removed if the minimum genotype quality <50, per cent alternate allele <0.8, or depth <10 using a Python script (https://github.com/broadinstitute/broad-fungalgroup/blob/master/scripts/SNPs/filterGatkGenotypes.py), and the number of these flagged sites for each sample was reviewed manually for any outliers with extreme numbers of filtered sites in any category (none were observed).

Alignment metrics were calculated on each sample BAM file using Picard v1.782. For phylogenetic analysis, sites with an unambiguous SNP (*n*=222,619) in at least one isolate were output in a FASTA file using a custom script (https://github.com/broadinstitute/broad-fungalgroup/tree/master/scripts/SNPs/vcfSnpsToFasta.py). The number of pairwise differences between isolates was calculated from the FASTA alignment using mega v7 to create the matrix file [24]. Maximum-likelihood phylogenies were constructed using RAxML v8.2.4 [25] using the GTRCAT nucleotide substitution model and bootstrap analysis based on 1000 replicates. Maximum-parsimony phylogenies were inferred using PAUP v4a164 [26].

### Pipeline 04 – NASP/GATK v3.7

Using NASP v1.1.2 [27], duplicated regions in the reference genome were masked with NUCmer (in MUMmer v3.23) [28]. Trimmomatic v0.32 [19] was used to remove sequencing adapters and to trim reads when average phred score dropped ≤20 within a 5 base sliding window. Reads with a remaining length ≥80 bases were aligned to the reference genome with BWA mem v0.7.15-r1142 [21]. SNPs were identified with GATK UnifiedGenotyper v3.7 [29] with no downsampling. SNPs in non-duplicated regions of the reference genome with ≥10× read coverage and ≥0.9 proportion consensus for every sample in the set were used for phylogenetic analysis. A consensus tree from the 40 most parsimonious trees by maximum parsimony was generated in mega v7 [24].

### Pipeline 05 – GATK V3.7.9 FireCloud/Terra

The GATK pipeline described above in dataset03 was reimplemented via Workflow Description Language (WDL) and deployed in the cloud-native platform Terra, (https://app.terra.bio/#workspaces/broad-fungal-firecloud/broad-fungal-gatk3) [30]. The indel realignment step was removed, as this step was no longer necessary after GATK v3.4. The WDL is also available in Github (https://github.com/broadinstitute/fungal-wdl/gatk3). The matrix construction and phylogenetic analysis were carried out as described for dataset 03.

### Pipeline 06 – GATK v3.8.0

The pipeline WGS-Outbreaker (https://github.com/BU-ISCIII/WGS-Outbreaker) was used for SNP identification. Read quality was assessed with FastQC v0.11.8 and reads were trimmed with Trimmomatic v0.32 with parameters ILLUMINACLIP:/opt/Trimmomatic-0.33/adapters/TruSeq3-PE.fa:2 : 30 : 10 TRAILING:10 SLIDINGWINDOW:4 : 15 MINLEN:70 [19] and aligned with BWA mem v0.7.12 [21]. SNPs were called using GATK HaplotypeCaller v3.8.0 (parameters: -stand_cal_conf 30 –emitRefConfidence GVCF -ploidy 1) followed by the GenotypeGVCF module with default parameters. SNPs were site filtered with the following criteria: depth <4, GQ <10.0, PL <20 and sequencing quality <50.0 following GATK best practice protocols [14, 22]. A phylogenetic tree was constructed using maximum-likelihood RAxML v8.2.9 (inference parameters: ‘-m GTRCAT -V -w’ with 100 bootstrap replicates).

### Pipeline 07 – GATK v4.0.6

The 35 samples were quality checked and filtered using FastQC v0.11.9 and Trimmomatic v0.35 [19]. The Illumina adaptors are concatenated to make a list of universal_Illumina adaptor. Trimming was performed in pairend mode with ILLUMINACLIP with universal_Illumina adaptor at seed mismatch 2, palindromeClipThreshold 30, simpleClipThreshold 10; LEADING:10 TRAILING:10 SLIDINGWINDOW:4 : 15 MINLEN:30. The processed reads were aligned to the reference genome (PEKT02000000) using BWA-MEM v0.7.17. The alignment was converted to binary alignment map and sorted using GATK SortSam. Subsequently, duplicate reads were marked and short variants were called through GATK HaplotypeCaller v4.0.6 in default setting – diploid mode [22]. No filtering was done. The sample wise variant call files were combined using bcftools v1.7 merge (http://www.htslib.org/doc/bcftools.html) and the whole genome for 35 samples was generated by incorporating variants found in the corresponding samples by using bcftools consensus.

The reference genome and draft genomes were generated for 35 isolates and concatenated in a single file for multiple sequence alignment using MAFFT v7.419 [31] in the default setting. A phylogeny was constructed using the maximum-parsimony method using mega v7 and the most parsimonious tree was reported. Pairwise SNP numbers between isolates were calculated from the multiple sequence alignment.

### Pipeline 08 – GATK v4.0.9

Sequencing reads were quality controlled using FastQC v0.11.8 (https://www.bioinformatics.babraham.ac.uk/projects/fastqc), and then trimmed using Trimmomatic v0.36 [19] with the parameters ILLUMINACLIP:TruSeq3-PE.fa:2 : 30 : 10 LEADING:3 TRAILING:3 SLIDINGWINDOW:4 : 15 MINLEN:36 and ILLUMINACLIP:TruSeq3-PE.fa:2 : 30 : 10 LEADING:3 TRAILING:3 SLIDINGWINDOW:4 : 15 MINLEN:100. The reference was indexed using BWA index v0.7.17-r1188, SAMtools faidx v1.7 and a dictionary constructed using GATK CreateSequenceDictionary v4.0.9.0. Sequencing reads were aligned to the reference using BWA mem v0.7.17-r1188 and sorted to BAM format using SAMtools v1.7 [12]. Sequencing reads were mark duplicated using Picard v1.8 and indexed using SAMtools. Variants were identified with GATK HaplotypeCaller v4.0.9.0 [22]. The obtained VCF file was used as -known-sites for GATK BaseRecalibrator to detect systematic errors in base quality scores. The obtained table was used to create a new BAM file containing recalibrated read data using GATK ApplyBQSR. The newly created BAM file was used create the final VCF file using GATK HaplotypeCaller. The SNPs were selected using GATK SelectVariants --select-type SNP v4.0 and filtered using GATK VariantFiltration v4.0.9.0 with the filter expression ‘QD <2.0 || MQ <40.0 || FS >60.0 || SOR>3.0 || MQRankSum <−12.5 || ReadPosRankSum <−8.0’. All of the VCF files containing filtered SNPs were merged using the VCF-merge tool into a common VCF file. The VCF file containing the filtered SNPs was converted to a GDS format using the function snpgdsVCF2GDS of the SNPRelate R package v0.9.19 [32]. The sequences of the final FASTA alignment were generated based on the GDS file using the function gds2fasta of SNPRelate. The phylogenetic tree was generated based on the final FASTA alignment using maximum-parsimony analysis determined by mega X v10.0.5 for Windows 64 platform with Tree-Bisection-Reconnection selected for MP search method, 10 as the number of initial trees, 1 as MP search level and 10 maximum trees to retain. To generate pairwise SNP numbers between samples, we used our own Python script (available at https://github.com/vuthuyduong/SNPanalysis/blob/main/scripts/computePairwiseSNPs.py), simply calculating the total number of different nucleotides between the sequences in the final FASTA alignment file. The analysis was also performed without the trimming step. This step slightly influenced the results, as the number of SNPs differed insignificantly (0.02 %).

### Pipeline 09 – SAMtools v0.1.19

Reads were aligned using with Stampy v1.0.23 (without Burrows–Wheeler aligner premapping, using an expected substitution rate of 0.01) [33]. Variant calling was undertaken using SAMtools v0.1.19 with base alignment quality (BAQ) enabled. Repeat regions were masked and SNPs were not called within these. These regions were identified using a self-blast approach (https://github.com/davideyre/bug-flow/blob/master/bin/genRefMask.py). To pass quality filtering, SNPs identified were required to have a quality >30, to be homozygous under the diploid model, demonstrate >75 % of bases in agreement with the consensus call and have a minimum depth of 5, with at least one read in each direction. Quality filters were determined based on previous repeat sequencing of the same bacterial isolates, targeting a false SNP rate of less than 1 per 100 Mb of reference genome called, i.e. prioritizing specificity in settings where WGS is used to exclude the possibility of transmission. Phylogenetic trees were constructed by merging input FASTA files and setting all invariant sites with missing data to match the reference for computational efficiency. An initial maximum-likelihood tree was constructed using PhyML v20 120 412 using the ‘BEST’ topology search option and a GTR substitution model.

### Pipeline 10 – NASP/SAMtools v0.1.18

The reads were trimmed using Prinseq v0.20.3 (http://prinseq.sourceforge.net/) using the parameters -trim_left 15 -trim_qual_left 20 -trim_qual_right 20 min_len 100 min_qual_mean 25 -derep 14 and then processed using NASP pipeline [27] with the option job management set to none. NUCmer (MUMmer v3.23) [28] was run to mask duplicated regions in the reference genome. Reads were aligned with BWA-mem (BWA v0.7.7) [21]. Using the NASP pipeline, SNPs were called with SAMtools v0.1.19 [12], selecting positions in non-duplicated regions of the reference genome, with ≥10× coverage, and with ≥0.9 proportion consensus. Phylogenetic reconstruction was performed using the maximum-parsimony method in mega v7 [24].

### Pipeline 11 – NASP/Trimmomatic/SAMtools v0.1.18

Reads were processed using following options in NASP v1.0 [27] with the option job management set to none. NUCmer (MUMmer v3.23) [28] was used to mask duplicated regions in the reference genome. Reads were trimmed with Trimmomatic v0.35 [19] to trim adapter sequence and low-quality bases (with parameter SLIDINGWINDOW:5 : 20), retaining a minimum remaining read length of 80 nucleotides. Reads were aligned with BWA-mem (BWA v0.7.7) [21]. SNPs were called with SAMtools v0.1.19 [12], selecting positions in non-duplicated regions of the reference genome, with ≥10× coverage, and with ≥0.9 proportion consensus. Phylogenetic reconstruction was performed using the maximum-parsimony method in mega v7 [24].

### Pipeline 12 – Pilon v.1.9

Reads were aligned with BWA mem v0.7.4, converted to BAM with SAMtools v0.1.18 view and sorted with SAMtools sort, and paired read alignment reads were selected with SAMtools view with parameter -f 0 x2 [12, 21]. Pilon v1.9 [34] was run on this file, using -VCF to select VCF output using a minimum depth of 0.1 for the average depth per sample to call a site. Positions flagged as ‘LowCov’, ‘Amb’ and ‘Del’ were removed using grep. Next, ECA-maker.pl was used to save all reference bases and SNPs from the VCFs, find positions that were present in all (entirely covered in all; ECA) and different in at least one, and print out those positions in FASTA and tabulated format. The FASTA was converted to NEXUS format (FASTA-parser.pl) and a parsimony tree was inferred using PAUP v4.0b10 [26] (PAUP commands:exe ca.nex; set autoclose=yes; set criterion=parsimony; set storebrlens=yes; set increase=auto; hsearch addseq=random nreps=1000 swap = tbr hold = 1; savetrees file=ca.nex.parsimony.tre format=altnex brlens=yes). ECA-maker.pl and FASTA-parser.pl scripts are available on Github (https://github.com/rhysf/ECATools).

### Pipeline 13 – SAMtools v1.3.1

In this pipeline, raw reads were analysed without trimming or analyses of base call quality. Paired reads were aligned to the reference genome using BWA aln and BWA-sampe (BWA v0.7.15-r1140) under default parameters and the intermediate files were converted to BAM using SAMtools view (SAMtools v1.3.1) with -bS options. The resulting BAM files were sorted and indexed using SAMtools sort and index commands with default parameters. Next, VCF files were generated using SAMtools mpileup with -uf options. Then SNPs were called using bcftools call (bcftools v1.3.1) with the --ploidy 1 and -c options, and viewed using the bcftools view command. The resulting files were piped to SAMtools vcfutils.pl script with the maximum read depth filtered using the varFilter -D 200 option. A series of custom Python scripts (available at https://github.com/jessieuehling/GWAS_scripts) were used to parse the results into a SNP table (MergeSNPs.py), and modify that SNP table into a FASTA file (SNP_fasta.py) for phylogenetic analyses.

### Pipeline 14 – Pathogenwatch

In this pipeline, individual FASTQ files (56 files representing 35 isolates) were handled separately. Reads were trimmed with Trimmomatic: trimmomatic-0.32.jar PE -phred33 LEADING:3 TRAILING:3 SLIDINGWINDOW:4 : 15 MINLEN:50 [19]. Annotated assemblies were produced from the trimmed reads using the pipeline described in [35], except that multiple assemblies for each sample were also generated with SPAdes v3.10.0 [36], with the k-mer sizes 41, 45, 49, 53, 57, 61, 65, 69, 73, 77, 81, 85, 89, 93, 97, 101, 105, 109, 113, 117, 121 and 125. An assembly improvement step was applied to the assembly with the best N50 and contigs were scaffolded using SSPACE [37] and sequence gaps were filled using GapFiller [38]. Automated annotation was performed using PROKKA v1.5 [39] and a genus-specific database from RefSeq [40]. SPAdes assemblies were selected for downstream analyses based on the assembly quality metrics.

Pathogenwatch is a web application that supports SNP-based neighbour-joining trees for *C. auris* inferred using a curated core gene library comprising 4250 genes as of September 2022. Single-copy orthologues were identified with OrthoMCL v1.458 (Markov index 1.5; maximum e-value 1e−5) using one representative annotated genome assembly for each clade using (B8441, B11220, B11221 and B11243). Assemblies were uploaded to the website via the upload page https://pathogen.watch/upload) and queried against the core library of representative genes with blastn v2.2.30. Pairwise distances between assemblies were scored by comparing all variant positions from all pairs of core-gene sets, removing loci that show an unusually large (or small in more distant comparisons) number of variant sites, counting SNPs (generating a downloadable pairwise difference matrix) and normalizing by the relative proportion of the core present in each assembly (generating a downloadable pairwise score matrix). The pairwise score matrix was then used to infer a midpoint-rooted neighbour-joining tree, as described in more detail in the documentation (https://cgps.gitbook.io/pathogenwatch/technical-descriptions/core-genome-tree). The Pathogenwatch collection of this dataset can be explored here: https://pathogen.watch/collection/573n97bw1rn4-c-auris-cdc-pilot-core-4250. Assemblies were also queried with blastn v2.2.30 for the presence of single point mutations known (as of September 2022) to confer resistance to fluconazole, 5-flucytosine, anidulafungin, caspofungin and micafungin.

### VCF standardization and summarization

Of the 14 datasets (e.g. results obtained from 14 pipelines), a subset of 12 (datasets 1–12) provided valid VCF files and were thus used for downstream comparison. To compare these VCF files, we standardized the sample order, sample names and contig names. Sample names were standardized to CA01-CA35 and were ordered lexicologically. Contig names were standardized to scaffold00001-scaffold00015. We removed sites that were not SNPs and not flagged as PASS, with heterozygous genotypes and non-canonical characters in ALT or REF fields (such as asterisk marks, dashes, underscores or points) from the VCFs. We also removed unused ALT alleles and standardized diploid genotypes to haploid (changed genotypes of 1/1 to 1). For ease of analysis, minimal versions of the VCFs were also produced by removing all INFO fields and non-GT FORMAT fields. VCF files were cleaned and compared using code available as a supplementary IPython notebook (https://github.com/broadinstitute/isham_wgs). Matrix files were compared using the R notebook code provided as a supplementary file (Data S1). All subsequent analyses were performed on the minimized VCFs. Summary statistics, such as allele frequency distributions, missingness distributions and site number were calculated by bcftools v1.8 [12].

### Site concordance analysis

To compare the 12 call sets, site level concordance analysis was performed on the standardized VCFs of each dataset. Site level concordance was based on the presence of a site in the call set, regardless of the sample level information. The analysis was performed by merging the site-only VCF of each call set and calculating the frequency of each site using bcftools v1.8. Each site could be classified into 12 conservation categories based on its frequency in all call sets. Private SNPs were defined as sites that were only present in one call set. The quality of each category was evaluated in detail for two representative call sets, 7 and 12, by comparing the distributions of three metrics: map quality scores (MQ), quality by depth (QD) and total depth (DP).

### Sample level concordance analysis and generation of consensus VCF

Sample level concordance was evaluated across datasets for each sample. We implemented a function vcf.pairwise_concord to calculate the pairwise concordances of all samples. The implementation was benchmarked against the GATK GenotypeConcordance tool and was included in the Python 3 package funpipe (https://github.com/broadinstitute/funpipe), a package for fungal genomic pipeline development. To summarize SNP sites and genotype calls identified by most of the pipelines, we defined a consensus genotype as a genotype that was called in 10 or more call sets (out of the 12); we included sites missed by up to 2 pipelines based on the high overall frequency of sites supported by 10 or 11 pipelines. We produced a VCF including all consensus genotypes, hereafter referred to as the consensus VCF.

### Generation of truth SNP sets using genome assemblies

To evaluate the quality of each dataset, we generated a truth SNP set using chromosomal level assemblies of one sample from clade III, isolate B11221 (sample CA05; accession GCA_002775015.1 [18]), and one sample from clade IV, isolate B11245 (sample CA06; accession GCA_008275145.1 [41]). Each assembly was aligned to B8441 (PEKT00000000.2) using NUCmer (MUMmer v3.22) [28]. Alignments were filtered using delta-filter (MUMmer v3.22) with option -g to keep only the alignments that form the longest mutually consistent set. Then, SNPs variants were identified using show-snps (MUMmer v3.22) using option -C to exclude SNPs contained in repeats, ambiguous mapping or indels. The SNP file from NUCmer was reformatted as a VCF for downstream comparison.

### Evaluation of sensitivity and specificity of each pipeline

The truth SNP sets were used to evaluate the quality of each call set. The GATK GenotypeConcordance tool was used to access the sensitivity and specificity of each SNP calling method. Since not all pipelines called reference alleles, we considered all ‘unavailable’ categories reference calls. Sensitivity was defined as false negative/(false negative+true positive), while specificity was defined as 1−false positive/(false positive+true negative). The harmonic mean of specificity and sensitivity (F1 score) was calculated as 2×specificity×sensitivity/(specificity+sensitivity).

## Results

### Variant calling on the genome sequences of 35 *C. auris* isolates

Pair-ended WGS data from 35 *C*. *auris* isolates (CA01-CA35) was provided to all participant research groups to call variants using their preferred pipeline. Twenty-one isolates were sequenced with Illumina HiSeq2500, and 14 isolates were sequenced with Illumina MiSeq v2, which generatied paired 250 base reads on both platforms (Table S1). These samples were selected for having at least 40× sequencing coverage and representing each of the 4 major *C. auris* clades [5], including 20 samples from clade I, 4 samples from clade II, 3 samples from clade III and 8 samples from clade IV (Table S1). Control samples included pairs of samples from the same isolate (CA25 and CA26), the same patient (CA17 and CA18, also CA28 and CA29), or the same outbreak (CA08 and CA27) [1]. The B8441 isolate (CA30) representing a commonly used reference genome from clade I was also included as a control, as were two additional isolates, B11221 from clade III (CA05; accession GCA_002775015.1 [18]) and B11245 from clade IV (CA06; accession GCA_008275145.1 [41]), with chromosomal level assemblies. Sample identity, including clade assignment, was blinded to the participants. All groups used the B8441 PacBio assembly as the reference genome (PEKT00000000.2) [5, 18] for variant calling.

### Summary of variant discovery pipelines

A total of 14 SNP calling pipelines from 11 groups were evaluated. Groups employed different sequence quality control methods, variant callers and filtering criteria. Eight pipelines used quality trimming of reads prior to alignment. All pipelines used BWA for read alignment, with the exception of pipeline 9, which used the Stampy aligner. For variant calling, seven pipelines used GATK (six different versions, including two GATK4 and six GATK3), four used SAMtools (three versions) and the other three used either Pilon, CFSAN or an assembly-based method (Table 1). Among the pipelines using GATK, six used HaplotypeCaller and one used UnifiedGenotyper (pipeline 4). Variant filtering varied substantially, with pipelines requiring different criteria for read depth, percentage of reads matching the variant and inclusion or removal of repetitive regions of the genome.

**Table 1.** Summary of variant calling pipelines

ID	Group	SNP caller*	Ploidy †	Ref call	SNP only‡	Alignment	Trim reads	SNP removal criteria	Total SNP sites§	Multiallelic Sites	Heterozygous sites
**ds1-cfsan**	A	CFSAN	H	Y	Y	BWA v0.7.12 (MEM)	Y	minBaseQual: 0 minConsFreq: 0.6 minConsStrdDpth: 0 minConsStrdBias: 0	223 736	1503	0
**ds2-gatk3**	B	GATK v3.6 HC	H	N	Y	BWA v0.7.1	Y	FS >60.0 MQ <40.0 QD <2.0 DP <10	228 820	1346	0
**ds3-gatk3**	C	GATK v3.7.9 HC	H	Y	N	BWA v0.7.15 (MEM)	N	QD <2.0; FS >60.0 MQ <40.0 GQ≥50; AR ≤0.8 DP ≥10	221 724	1210	0
**ds4-gatk3**¶	D	GATK v3.7 UG (NASP)	H	Y	N	BWA v0.7.15	Y	<10× coverage <0.9 prop. consensus in duplicated regions	222 152	1161	0
**ds5-gatk3**	C	GATK v3.7.9 HC	H	Y	N	BWA v0.7.15 (MEM)	N	QD <2.0; FS >60.0 MQ <40.0 GQ≥50; AR ≤0.8 DP ≥10	221 790	1214	0
**ds6-gatk3**	A	GATK v3.8.0 HC	H	Y	Y	BWA v0.7.12 (MEM)	N	DP <4 GQ <10.0 PL <20 Seq. qual. <50.0	222 170	1267	0
**ds7-gatk4**	E	GATK v4.0.6 HC	D	N	N	BWA v0.7.17	Y	No filtering performed	224 812	1205	12 465
**ds8-gatk4**	F	GATK v4.0.9 HC	D	N	Y	BWA v0.7.17 (MEM)	Y	QD <2.0; MQ <40.0 FS >60.0; SOR >3.0 MQRankSum <−12.5 ReadPosRankSum <-8	220 197	1164	3903
**ds9-samtl**	G	SAMtools v0.1.19	D	Y	Y	Stampy v1.0.23	N	Qual >30 >0.75 consensus Min DP >5 (1 read)	202 854	998	0
**ds10-samtl**	H	SAMtools v0.1.18 (NASP)	D	N	N	BWA v0.7.7	Y	<10× coverage <0.9 prop. consensus in duplicated regions	216 782	1140	14 983
**ds11-samtrm**	H	SAMtools v0.1.18 (NASP)	D	N	N	BWA v0.7.7 (MEM)	Y	<10× coverage <0.9 prop. consensus in duplicated regions	230 151	1328	9325
**ds12-pilon**	I	Pilon v1.9	D	Y	N	BWA v0.7.4 (MEM)	N	Remove low coverage Amb Dels	228 121	1243	0
**ds13-samtl**	J	SAMtools v1.3.1 bcftools v1.3.1	D	na	N	BWA v0.7.15 (ALN)	N	Max. read DP >200	na	na	na
**ds14-pthwtch**	K	Pathogen-Watch	na	na	Y	na	Y	na	na	na	na

*HC, Haplotype Caller; UG, UnifiedGenotyper.

†H, haploid; D, diploid.

‡Pipeline only called or provided SNPs and no indels.

§Total SNP sites include multi-allelic and heterozygous sites.

¶Filtering criteria for pipeline 4 was not applied to the submitted VCF, but was used in generating the concatenated SNP alignment for phylogenetic analysis.

Each group provided the methods for their pipeline and an output dataset, including a VCF file of their variant calls, a matrix file of the number of pairwise SNP differences between samples, a whole-genome FASTA alignment and a phylogenetic tree inferred from the SNP calls. Insertions and deletions were reported in eight of the datasets, but they were not further evaluated due to the scope of this study. For ease of description, we used the order and abbreviation of the variant calling method to identify each dataset (Table 1, column 1). Among the 12 pipelines that produced standard VCF files (datasets 1 to 12), 6 called variants in haploid mode and the rest called in diploid mode. Of the seven GATK pipelines that incorporated reference calling, four (pipelines 3 to 6) performed joint calling. Site statistics were not calculated for dataset 13 due to irregularities in the VCF format and for dataset 14 due to sequencing lane level rather than sample level variant call (see Methods). To allow comparisons of SNPs identified by each pipeline, VCF standardization and filtering were carried out. Briefly, samples were ordered numerically, contigs were renamed uniformly, and indels, non-PASS SNPs, heterozygous genotypes, non-canonical genotypes (e.g. asterisk marks, dashes, underscores or points) and unused ALT alleles were removed. The final set of genotypes were standardized to haploid (i.e. 1 instead of 1/1) (see Methods).

### Summary and comparison of submitted SNP call sets

The total number of SNPs discovered by each pipeline ranged from 202 854 (pipeline 9) to 230 151 (pipeline 11), with an average of 221 942 SNPs (sd=7126) (Table 1). Of the six call sets that used diploid settings, four included thousands of heterozygous calls (3903 to 14 983, 2 to 6 % of sites respectively, Table 1), which are all false positive calls, as the *C. auris* genome is haploid [18, 42]. The total multi-allelic SNP sites discovered ranged from 998 (pipeline 9) to 1 503 (pipeline 1) (Table 1). In the provided multi-sample VCFs, missing positions not called in a given sample are largely explained by a lack of reference calling by some pipelines. Pipelines with reference calls (datasets 1, 3, 4, 5, 6, 9 and 12) tend to have a low number of missing positions, while the rest have a high number of missing positions (Fig. S2).

**Fig. 1.**
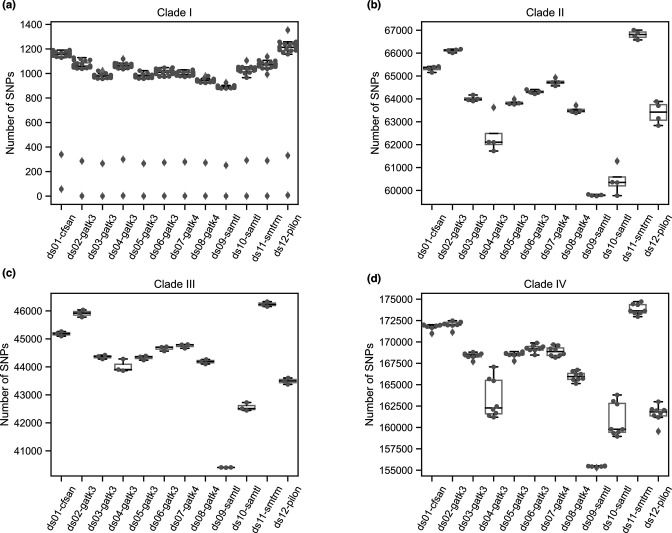
Single-nucleotide polymorphisms (SNPs) called by each pipeline per clade. Each plot depicts the total number of SNPs identified for each pipeline (datasets 1 to 12). The 35 samples are summarized in 4 plots by clade. (**a**) Clade I (*n*=20), (**b**) clade II (*n*=4), (**c**) clade III (*n*=3) and (**d**) clade IV (*n*=8).

As there is high variability between the clades of *C. auris* but low variation within clades [5], we next summarized the number of SNPs discovered within each clade (Fig. 1). The average number of SNPs in each clade was lowest in clade I, as the reference genome is also from this clade. We found high variation in the number of SNPs within each clade across datasets. Comparison of these numbers suggests that pipelines 9 and 10 identified fewer SNPs in clades II, III and IV compared to other pipelines, while pipelines 4 and 12 identified fewer SNPs in clades II and IV, and pipeline 11 identified more SNPs in clades I and IV compared to other pipelines. While these comparisons between pipelines can identify major differences in the datasets produced by each, there is not a gold standard dataset for these samples that can be used to calculate the accuracy of each method.

### Evaluation of specificity and sensitivity of SNP calling pipelines

**Fig. 2.**
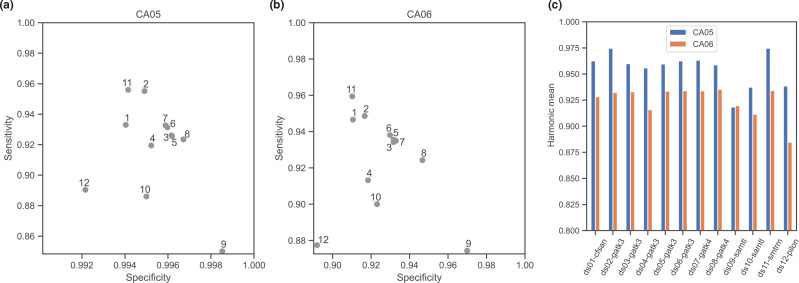
Sensitivity, specificity and their harmonic mean of each pipeline. Panels (a) and (b) depict the sensitivity (*y-*axis) and specificity (*x-*axis) for each pipeline (pipelines 1 to 12). Specificity and sensitivity were calculated by comparing variant calls from CA05 (B11221; clade III (a) and CA06 (B11245; clade IV) (b) to the truth set of SNPs identified between genome assemblies of these isolates with B8441 (clade I). (c) Barplot shows the distribution of the harmonic mean (F1 score) for sensitivity and specificity for each pipeline.

To evaluate the specificity and sensitivity of each pipeline, we generated an orthogonal call set for two samples, CA05 (B11221) and CA06 (B11245), by aligning genome assemblies of these isolates to the B8441 reference used for variant calling. These genomes for B11221 and B11245 were previously assembled to the chromosomal level using long-read sequences (PacBio or Oxford Nanopore, respectively) [18, 41]. In total, 47 251 and 176 126 SNPs were identified for CA05 and CA06, respectively, reflecting that the clade III CA05 isolate is more closely related to the clade I B8441 reference than the clade IV CA06 isolate [3, 5]. Using these SNPs as a truth set, we calculated that the average sensitivity across the 12 pipelines was 0.919 in CA05 and 0.924 in CA06. Pipeline 11 had the highest sensitivity (0.956 in CA05 and 0.959 in CA06), and pipeline 9 had the lowest sensitivity (0.850 in CA05 and 0.874 in CA06). To calculate specificity, since not all pipelines called reference alleles, we treated the missing genotypes of each dataset as reference alleles (see Methods). This assumption may overestimate the true specificity. The average specificity was 0.995 in CA05 and 0.926 in CA06. While almost all pipelines showed high specificity with CA05, their specificities had higher deviation for the more divergent isolate CA06. Pipeline 9 showed the highest specificity in CA06 (0.97), while pipeline 12 showed the lowest specificity in CA06 (0.892) (Fig. 2). Combining the sensitivity and specificity measures using the harmonic mean, we found an average value of 0.955 for CA05 and 0.925 for CA06. For CA05, the highest harmonic mean was found for pipeline 2 and the lowest was for pipeline 9. For CA06, the highest harmonic mean was found for pipeline 8 (0.935) and pipelines 5, 6, 7 and 11 (0.934) and the lowest was for pipeline 12 (0.885) (Fig. 2). These differences highlighted that different pipelines have tradeoffs between sensitivity and specificity that were impacted on by the genetic difference from the reference genome.

We assessed the impact of different pipelines and filtering criteria using this measurement of sensitivity and specificity for each pipeline. Overall, GATK methods (pipelines 2 to 8) balanced achieving high sensitivity and specificity relative to the other variant calling methods (Fig. 2). Many of the GATK-based pipelines clustered together (3, 5, 6 and 7), as their parameters and filtering criteria reflected the GATK best practices [14]. However, the GATK pipeline using UnifiedGenotyper (pipeline 4) showed lower sensitivity and specificity related to GATK pipelines using HaplotypeCaller. The sensitivity of this UnifiedGenotyper pipeline was also lower than that of those using SAMtools (e.g. pipeline 11), suggesting that this earlier version of GATK had a lower performance for this dataset. In addition, we observed that one of the factors that might impact on sensitivity and specificity was read trimming. Two NASP-based pipelines that use SAMtools to call variants processed reads with different trimming software (pipelines 10 and 11) and this resulted in differences between the SNP calls. We observed a large sensitivity difference (5 –7 %) between pipelines 10 and 11 (0.886 vs 0.956 in CA05 and 0.900 vs 0.959 in CA06), where the only difference between these pipelines was the trimming software (Prinseq in pipeline 10 and Trimmomatic in pipeline 11), highlighting the impact of differences in trimming on SNP calling in the SAMtools-based pipelines. Meanwhile, the specificity between the two pipelines remained similar (0.995 vs 0.994 in CA05 and 0.923 vs 0.910 in CA06). In contrast, GATK-based pipelines appeared to be less affected by read trimming (e.g. comparing pipeline 7, which used trimming, with pipelines 3, 5 and 6, which did not carry out trimming).

### Unique and shared SNPs across call sets

**Fig. 3.**
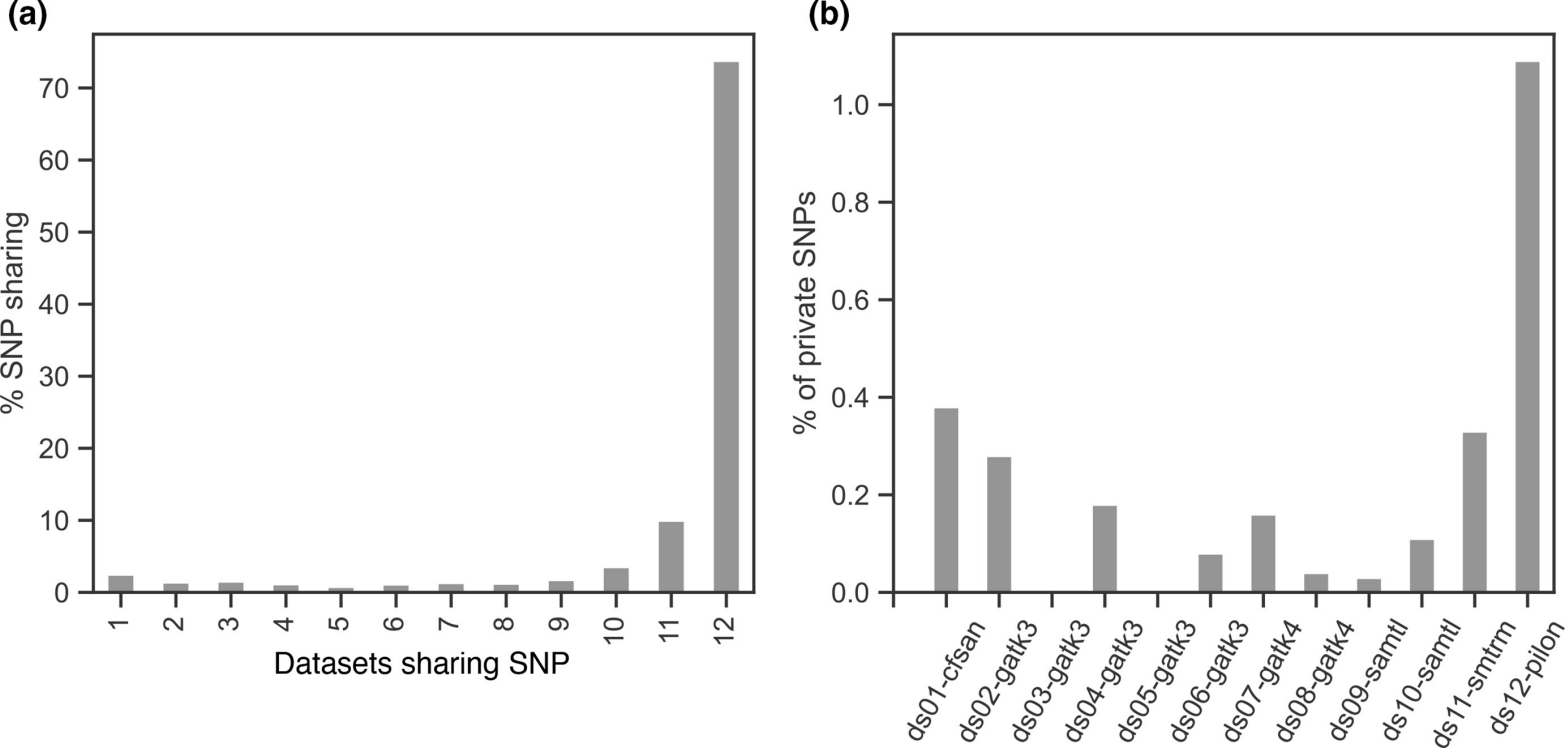
Comparison of SNPs called across datasets from 12 pipelines. (**a**) The percentage of all detected sites called by as few as just 1 to as many as all 12 pipelines is shown. (**b**) The percentage of all sites that represent private SNPs is shown for each pipeline.

To compare SNP call sets across pipelines, we evaluated the sites discovered in datasets generated by all pipelines (common SNPs) or only one of the pipelines (private SNPs). A total of 75 % of all SNPs were found in all datasets and 87 % of sites were common across 10 or more datasets (Fig. 3a). Most private SNPs were in the dataset from pipeline 12, which used Pilon (Fig. 3b). A major reason that the number of private SNPs was higher in this dataset is likely that the underlying method was only run in this one pipeline, as compared to GATK and SAMtools, which were used in several pipelines.

**Fig. 4.**
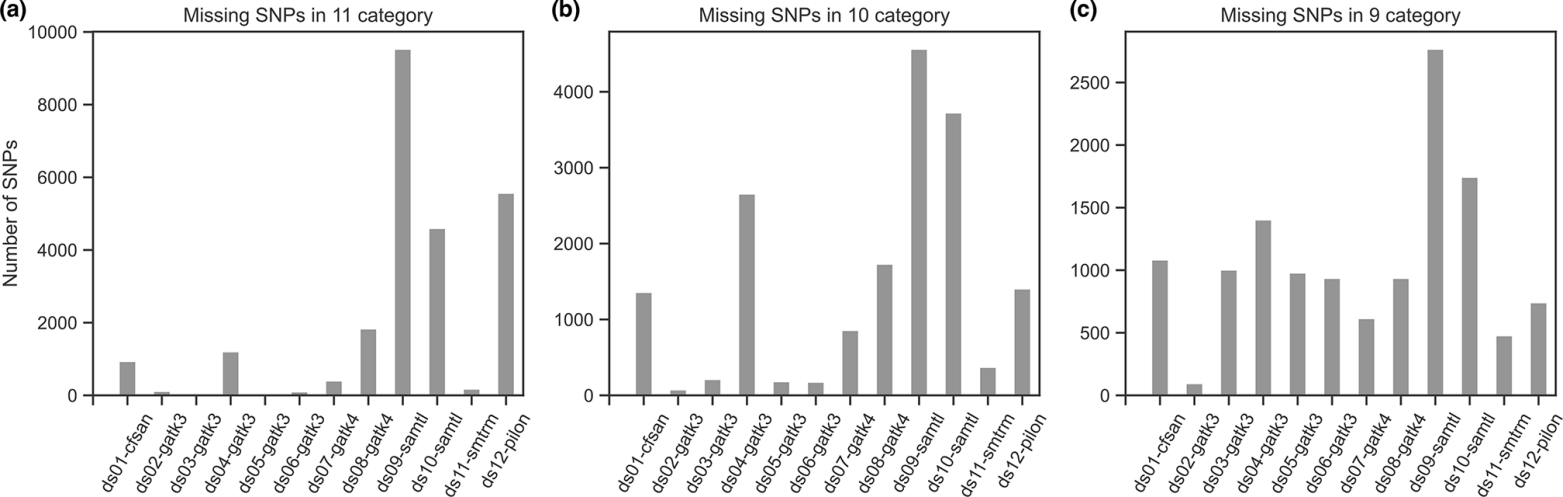
High-confidence SNPs missed by each pipeline. For sites found in (**a**) 11, (**b**) 10, or (**c**) 9 datasets, the number missed by each pipeline is summarized.

We then evaluated which pipelines missed the SNPs found by nearly all other pipelines, which likely represent high-confidence sites. We tabulated which pipelines missed a call that was found in 11, 10 and 9 other datasets (Fig. 4). For the 11 category (missed in one), pipelines 9, 12 and 10 missed the highest number of SNPs. For the 10 category (missed in two), pipelines 1, 4, and 8 had the next highest frequency of missed SNP calls. For the 9 category, every pipeline showed evidence of missing SNPs. This suggests that sites found in 10 or more datasets could be used to define a high-confidence SNP category that any pipeline should discover.

To better understand the features of private SNPs, we evaluated the quality of those SNPs using dataset 7 and dataset 12, as they included quality annotations. We examined the distribution of the map quality score (MQ), quality by depth (QD) and depth (DP) for each SNP sharing category. While SNPs that were identified by all pipelines showed high quality and depth, we found that as the number of pipelines that identified each particular SNP decreased, the quality metrics of this SNP also decreased, with more sites showing lower MQ, QD and DP values (Fig. S3). This confirmed that most private SNPs were of lower quality and represented borderline SNP calls.

### Unique and shared SNPs amongst call sets for each isolate

**Fig. 5.**
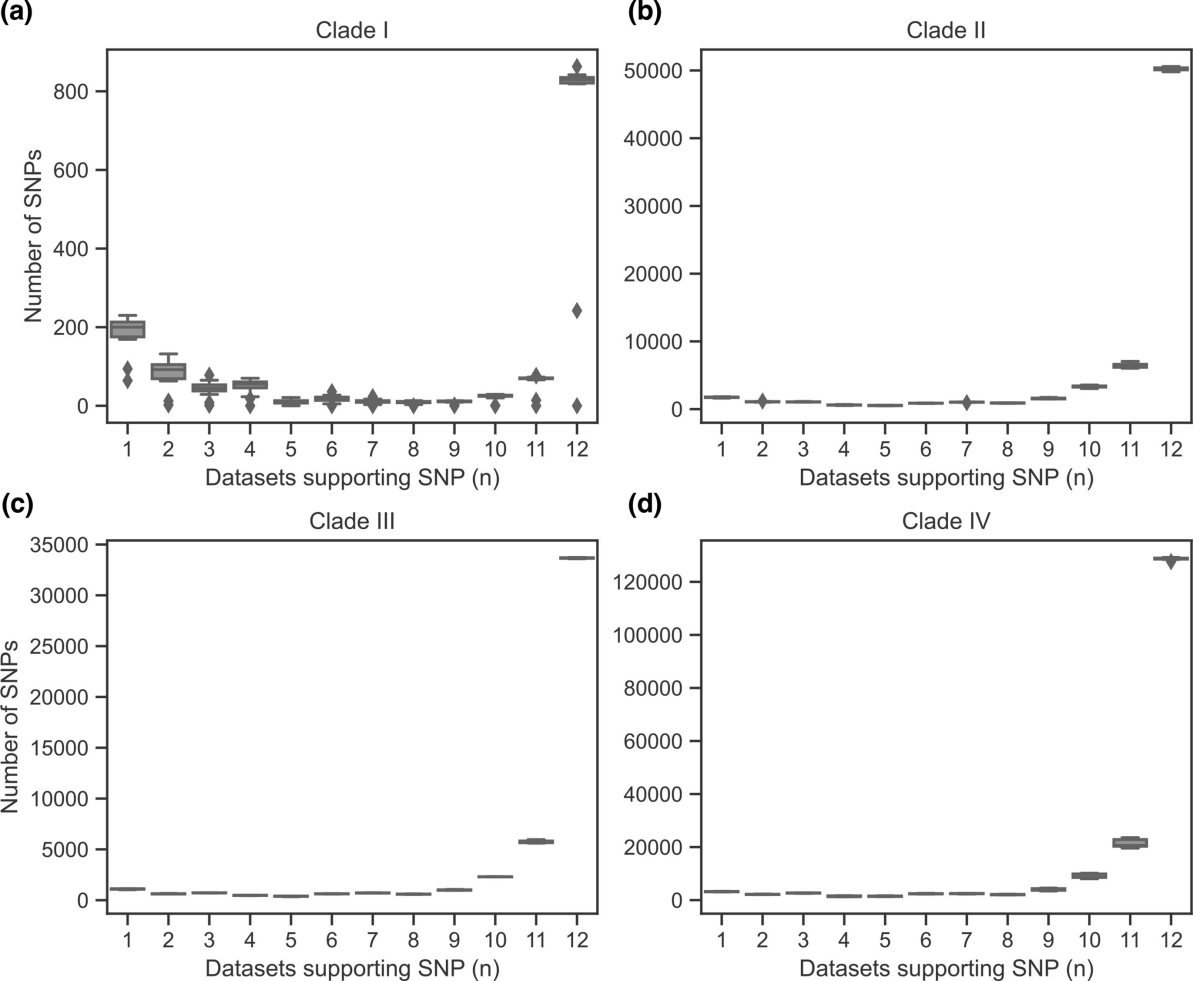
Sample level SNP concordance by clades. Sites were compared for each sample across the datasets produced by 12 pipelines, and the number of pipelines supporting each SNP is shown by clade. The number of SNP sites identified for each sample in between 1 and 12 datasets is summarized by clades: (**a**) clade I (*n*=20), (**b**) clade II (*n*=4), (**c**) clade III (*n*=3) and (**d**) clade IV (*n*=8).

After comparing SNP site discovery across pipelines, we examined SNPs at the isolate (sample) level. For each isolate, we calculated the number of the 12 pipelines that discovered each SNP and aggregated these counts for all isolates in a given clade (Fig. 5). By examining how well a SNP call for a given isolate was reproduced across all datasets, we found that there was an overall high SNP concordance with a very low frequency of SNPs called by fewer than nine pipelines. Comparing the four clades, clade I isolates – which had fewer SNPs overall because they showed the greatest similarity to the reference genome – had a higher proportion of sites called by only one or two pipelines (Fig. 5a). We next examined the source of private SNPs, focusing on those called by a single pipeline. We found that a higher proportion of private SNPs in clade I were predominantly found in datasets 1 and 12 that also identified a higher number of private SNPs in other clades (Fig. S1). The higher proportion of private SNPs in clade I likely appears amplified because of the smaller number of SNPs called for clade I compared to other clades. For clades II, III and IV, the highest number of private SNPs at the sample level was called in dataset 12 (Fig. S1), consistent with the site level analysis (Fig. 3). Using the results of this analysis, we defined a high-confidence SNP call set of the sample level genotypes discovered in at least 10 datasets.

### Similarities of SNPs discovered from the same isolate, patient or outbreak

To examine the effect of different variant calling methods on inferring the genetic relationship of closely related isolates, we included three pairs of control samples that came from the different DNA extractions of the same isolate (CA25 and CA26), same patient (CA28 and CA29) or same outbreak (CA08 and CA27). We expected that SNPs predicted from the same isolate should be identical, and those from the same patient or same outbreak should show roughly 3–7 SNP differences on average [15]. Except for pipelines 4 and 6, all other pipelines, discovered a low number of unique SNPs between isolates from each pair (between 0 and 6; Table 2). For pipeline 4, the false-positive SNP calls between these control samples are likely explained by the lack of filtering applied to the submitted VCF, as filtering was only applied in the process of inferring a phylogeny in this pipeline. Even though most pipelines identified low numbers of unique SNPs, in some pipelines this came at the expense of missing some shared SNP sites (i.e. SNPs called in both of the pair of isolates relative to the reference). For example, pipeline 9 found few SNP differences between closely related isolates, but discovered a lower number of shared SNPs, suggesting that the filtering parameters were over-stringent in eliminating these SNP sites reproducibly called by other pipelines (Table 2). In addition to the isolates from the same patient or outbreak, a sample matching the reference genome was also included as a control (CA30). Most pipelines found 0 or 1 SNP between CA30 and the reference genome. However, pipeline 4 found 3 sites, pipeline 12 found 7 sites, and pipeline 1 found 57 sites. These variable SNP sites found between CA30 and the reference most likely represent false-positive SNP calls.

**Table 2.** SNP call comparisons based on VCF for each pipeline for control isolate comparisons

Group	Unique SNPs			Common SNPs			Reference
	Isolate*	Patient†	Outbreak‡	Isolate*	Patient†	Outbreak‡	B8441§
**ds01-cfsan**	5	4	6	1135	1147	1142	57
**ds02-gatk3**	0	0	0	1020	1065	1027	1
**ds03-gatk3**	1	4	2	955	974	961	0
**ds04-gatk3**	33	24	24	1027	1053	1024	3
**ds05-gatk3**	1	4	2	956	977	961	0
**ds06-gatk3**	23	26	31	974	1010	980	1
**ds07-gatk4**	0	0	0	971	1000	990	1
**ds08-gatk4**	0	0	0	923	948	942	1
**ds09-samtl**	0	3	2	878	894	894	0
**ds10-samtl**	0	0	0	991	1031	1011	0
**ds11-smtrm**	0	0	0	1025	1073	1042	1
**ds12-pilon**	1	3	3	1146	1176	1182	7

*Same isolate, CA25 and CA26.

†Same patient, CA17 and CA18.

‡Same outbreak, CA08-CA27.

§B8441 (CA30), the reference genome used for read alignment.

### Discovery of drug resistance mutations in *ERG11*

As WGS analyses are increasingly used for identification of drug resistance mutations, we evaluated whether all pipelines (including Pathogenwatch) were able to discover point mutations associated with azole resistance in *C. auris*. In azole-resistant isolates, known point mutations (K143R, Y132F, or F126L) in the gene encoding the lanosterol-14α-demethylase enzyme targeted by the triazoles (*ERG11*) were correctly identified by all pipelines. In susceptible isolates lacking these variants (i.e. wild-type genotype as the reference genome), a reference allele was reported by the subset of pipelines that called the reference alleles, whereas pipelines that did not include reference calling did not report a genotype at these positions.

### FASTA alignment comparison

We also evaluated the FASTA alignment of whole-genome variants generated from VCF files to identify differences that could be attributed to the SNP calling protocol or additional processing steps used to create alignment files. FASTA alignments were submitted for 13 pipelines (1 to 13). Half of the alignments included a reference genome call. Only pipeline 7 included invariant sites, gaps and ambiguous nucleotides. For consistency, these positions were stripped from the alignment to only include positions that vary in at least one isolate. The average length of the FASTA alignment in all 13 datasets was 213 837 bases (sd=18 471), 8105 fewer than the average number of SNPs discovered by each pipeline from VCF files. Pipelines 4, 7,10, 11 and 12 excluded more than 9 % of variants sites (30 210 on average) in the FASTA alignment relative to the total SNPs discovered. This suggests that some pipelines removed a large proportion of the SNPs during conversion of the VCF to a multi-FASTA alignment.

### Comparison of pairwise SNP matrices

**Fig. 6.**
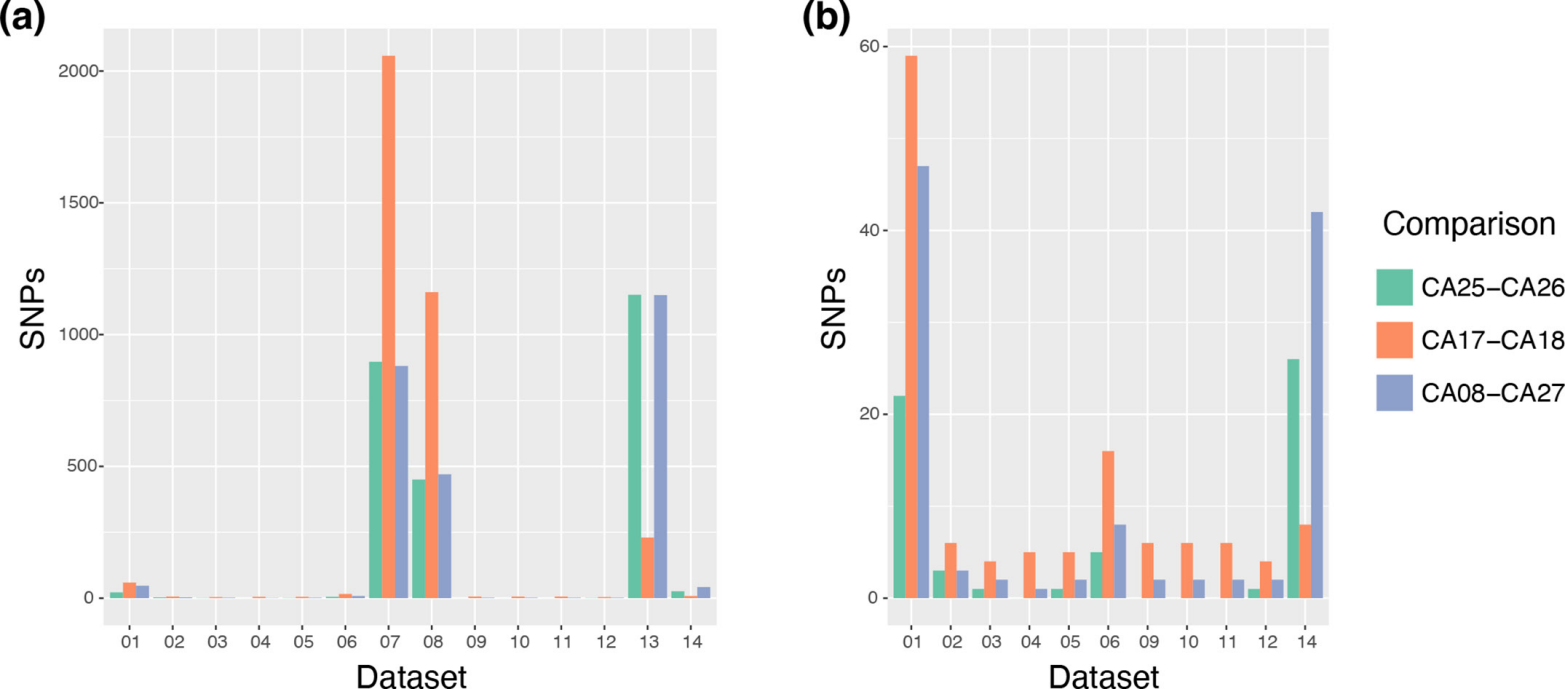
Pairwise differences between control pairs of isolates reported by each pipeline. (**a**) Pairwise differences reported in matrix files for all 14 pipelines. (**b**) Pairwise differences excluding pipelines with high reported differences (7, 8 and 13).

Pairwise differences in the number of SNPs from whole-genomic data are often used to estimate the level of genetic variation between isolates. To examine the impact of variant calling pipelines on counts of SNP differences, each group submitted a matrix of the counts of pairwise SNP differences. While 14 pipelines submitted a matrix, pipeline 14 examined variants at the sequencing lane level rather than the sample level; only counts from the first lane of each sample with multiple sequencing lanes were used to compare results. Three datasets (7, 8 and 13) reported high rates of SNP differences between the three pairs of control samples (Fig. 6). Inspecting the datasets with lower numbers, datasets 1 and 14 had elevated SNP counts compared to the others. We also summarized the number of differences found within or between samples from each clade. Here also, datasets 7, 8 and 13 appear to be outliers, with higher numbers in 7 and 8 and very large variability in the differences reported by 13 (Fig. 6). These comparisons between clades also highlighted that datasets 4 and 14 had fewer differences reported compared to the other datasets, suggesting that these pipelines may underestimate genetic variation. As SNP matrices were typically produced from the FASTA alignment, additional filtering steps in the generation of the FASTA file can affect these counts.

### Concordance of phylogenetic trees

**Fig. 7.**
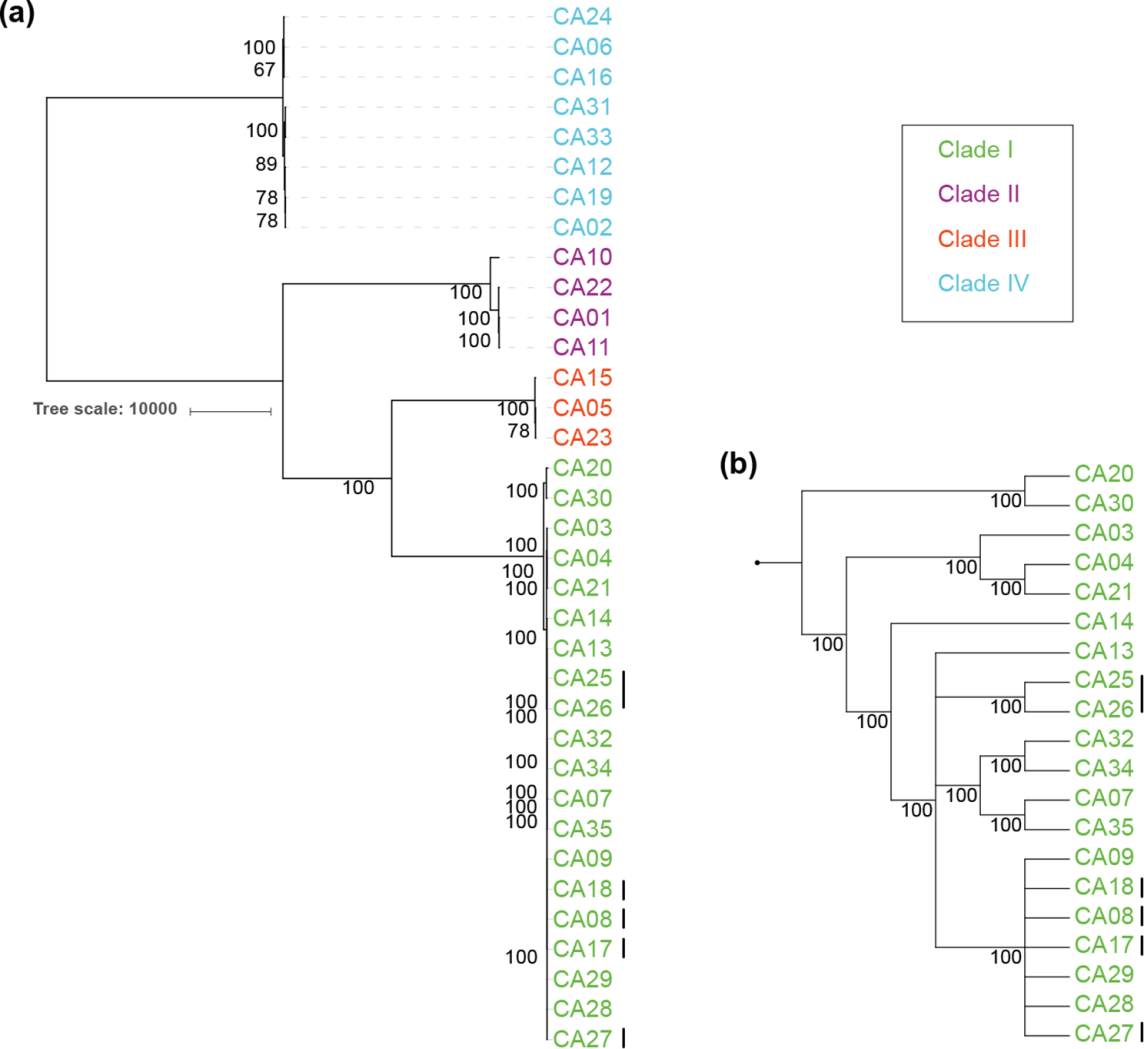
Consensus tree from maximum-parsimony trees generated by each pipeline. Consensus support across trees provided for 10 pipelines is shown for nodes with at least 50 % consensus support for all isolates (**a**) and for clade I isolates (**b**). Nodes without support have taxa disagreement between the trees from different pipelines. Taxa labels (CA01–CA35) are coloured by clade (legend). Vertical lines next to taxa labels indicate control sample pairs shown in Fig. 6.

Phylogenetic trees generated using maximum parsimony were submitted for 10 datasets. Half of them included a reference genome call and half did not report the reference. To compare the phylogenetic trees, the reference genome taxa were pruned for all trees to have 35 sample taxa. Consensus trees were generated using Consense (Phylip) with a 50 % support threshold. The overall topology of four major clusters is conserved in all trees (Fig. 7). Some samples in clade I and IV that have a very low numbers of SNPs show branching disagreement between the maximum-parsimony trees. The phylogenetic tree depicts the consensus support from 75–100 % in each node (branch labels; size scale in Fig. 7).

## Discussion

**Fig. 8.**
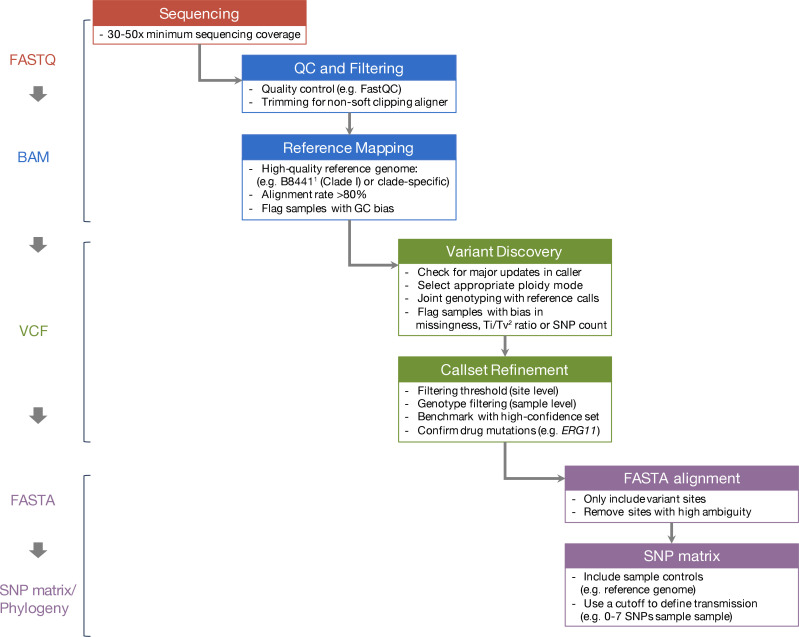
Workflow and recommendations for genomic variant identification protocols in fungi. The workflow is divided into four main colour-coded sections that are meant to be performed sequentially. Recommendations are listed within each step. ^1^The B8441 *C. auris* genome assembly is available in the *Candida* Genome Database (http://www.candidagenome.org) and NCBI (PEKT00000000.2). ^2^Ti/Tv ratio, Transition/Transversion ratio.

Accurate detection of variants in the genomes of microbial pathogens is an important prerequisite for understanding transmission patterns during outbreaks and studying the molecular evolution of fungi. Due to an increasing incidence of drug resistance among fungi and outbreaks of fungal diseases, and in parallel the expanding volume of available WGS data, there is an urgent need for consistent data analysis pipelines across different research groups. To this end, we assembled a consortium of research groups working on fungal pathogens from 11 research institutes in 6 countries, across 4 continents, to evaluate the similarities of individually developed SNP calling pipelines. We generated a set of WGS data from 35 *C*. *auris* isolates and performed a systematic comparison of SNP calls and phylogenetic trees from each group. Fig. 8 shows a summary of the workflow and the general recommendations drawn from this comparison. Recommendations are provided for the main steps in the analysis, including the importance of using quality control and high-quality references, establishing consistent filtering thresholds and benchmarking with high-confidence sets.

All isolates in our study were sequenced with at least 40× depth of coverage to ensure that pipelines had minimal areas of low coverage across the *C. auris* genome. We did not evaluate the effects of lower depths of coverage on pipeline performances, as this question was outside of the scope of our investigation, however lower sequencing targets may be suitable depending on the application. Many studies aim for 30–50× depth of coverage for WGS studies focused on SNP genotyping, but higher depths may be needed for genotyping organisms with more complex genomes or studies aiming to detect low-frequency variants [43]. However, this may benefit from fine tuning using truth sets in specific fungal species to optimize minimal coverage selection.

Overall, more than 80 % of the SNP sites were discovered by more than nine pipelines, and all pipelines were able to accurately reconstruct the phylogenetic relationships among isolates and accurately predict substitutions in *ERG11* linked to azole resistance. However, there were still large differences across call datasets in the predicted SNPs that could impact on the analysis of isolate relationships, including inferring transmission chains based on SNPs. We observed that differences in read trimming, SNP calling methods and downstream filtration steps contributed to the variation between the datasets generated by different pipelines.

Although variant calling methods using short-read sequencing data have matured during the last decade, the community has not yet reached a consensus on the best practices for SNP discovery in microbial genomes. Despite using different methods, most pipelines in this study were able to correctly identify most sites in the regions with good mapping qualities that were well represented in the sequence data and were not located in the repetitive regions of the genome. However, the agreement between datasets from different pipelines declined for sites located within regions with low mapping quality and depth of coverage, as these sites were removed by various read trimming and downstream filtering steps that varied between the pipelines. In selecting such parameters, users need to consider how their pipeline balances sensitivity and specificity, and this choice depends in part on the intended downstream analyses. In this study, several GATK-based pipelines performed well in both these categories, resulting in a high composite F1 score. While many GATK-based pipelines employed similar variant calling and filtration strategies, selected based on the GATK best practice recommendations [14], there has been little specific tuning of variant calling and filtering parameters for fungal genomes. In addition, the use of outdated methods (e.g. GATK UnifiedGenotyper) and settings (e.g. not using joint calling to inform variant calls) are not recommended and may have negatively affected the performance of some pipelines. Other factors to consider for pipeline comparisons include computational speed, requirements for resources and bioinformatics expertise. Although GATK-based pipelines demonstrated high sensitivity and specificity in our comparisons, these pipelines required considerable computational power and bioinformatic expertise to operate. However, incorporating these more advanced methods into easily accessible workflows running on the cloud, such as for the Terra GATK pipelines, addresses many of these needs in addition to ensuring that methods are reproducible.

Another parameter that had a large impact on SNP calling accuracy was the selection of the ploidy mode. Although most SNP calling methods were initially designed for diploid organisms, many now include a haploid option that should be used for *C. auris*. However, six pipelines used the diploid mode and did not filter sites with heterozygous calls from the submitted VCF of SNP calls, which resulted in a substantial number of the false-positive calls within these datasets (Table 1). While the correct selection of ploidy parameters is needed for accurate SNP discovery and to eliminate the need for additional downstream filtering, exploring the overall allele balance in a sample can also be a useful quality control step for detection of multiple isolates in a sample that might have originated from mixed infections or contamination.

Another factor affecting SNP calls was read trimming. In the WGS data used in this study, the second read of the read pair declined in quality across the read length (Fig. S3). While in theory, trimming should increase the quality of SNPs by removing low-quality positions, this step often removes sites found in difficult-to-sequence regions of the genome, decreasing the sensitivity of SNP detection; low-quality regions can be handled by SNP callers that allow for soft clipped bases that are not part of an alignment. The trimming method seemed to have a notable impact on the performance of a SAMtools-based pipeline (pipeline 10 vs 11), as switching from Trimmomatic to Prinseq greatly lowered the sensitivity. However, the performance of the GATK-based methods (pipeline 6 vs 3, 5 and 7) was not clearly affected by the use of Trimmomatic for read trimming. Additional features in GATK, such as joint genotyping, which is reported to help increase the precision in removing false positives and to increase the sensitivity in detecting low-frequency variants, may contribute to greater flexibility in handling read alignments.

All groups were able to reproduce the expected relationships among the four clades of *C. auris*, demonstrating that the clade assignment was able to tolerate some SNP disagreements. However, the relationships among isolates within each clade were affected by differences in the SNP calls, which affected the inferred phylogeny and conclusions about the relationship of isolates collected from the four major clades or from outbreaks. While excessive filtering would result in an inference of too close relationships, weak filtering could introduce false-positive SNPs and overestimates of divergence.

Although this study was not directly set up to evaluate the effect of different reference genome assemblies on SNP calls, the selection of reference genome can impact on the level of SNPs identified. While most studies have used a clade I reference genome, relying on alignments to a single reference may miss clade-specific or highly divergent regions present in other isolates. As complete genome assemblies have been generated for each clade [41], studies focusing on a set of isolates from a single clade, such as an outbreak, should consider selecting a reference genome from the same clade. In the future, variant calling methods that incorporate multiple reference genomes or use a reference-free approach could become more widely applied.

To evaluate the SNP calls, we developed an initial truth set for *C. auris*, using assemblies of two isolates from other clades in addition to the reference genome used for SNP identification. A richer collection of assemblies would increase the power for estimations of the sensitivity and specificity of each pipeline. An independent cross-validation set, such as Sanger sequencing or genotyping, would also be valuable to confirm selected sites. Some variant calling methods can utilize a curated truth set, such as the Variant Quality Score Recalibration function of GATK or DeepVariant, a deep learning based variant caller [44]; however, truth sets are not widely used for fungal variant calling. In human genomics, the Genome-In-A-Bottle project was developed to create a reference dataset for the community to use for benchmarking [45]. Generation of such ‘gold standard’ datasets for different fungal species would be very valuable for fine tuning variant calling methods for fungi. Recently, a whole-genome sequence benchmarking set was developed for *C. auris*, which includes well-characterized genomic reads from isolates from outbreaks from clade I [46]. As an additional benchmarking set, we generated a consensus SNP set, using sites and genotypes appearing in more than nine datasets. As we expect these will be found by nearly all pipelines, this consensus set for the 35 samples and the set of variants between the 3 genome assemblies could be used by others to benchmark any new SNP calling pipeline for *C. auris* and compare the results to those described here.

Global outbreaks have called for synergistic efforts in computational analysis of population genomics and genomic epidemiology in fungi, similar to parallel efforts in bacteria or viruses. Similar approaches have been started to be implemented in microbial pathogens to measure the performance of different pipelines [47]. As most groups in the fungal community use custom pipelines, moving towards a consensus pipeline or set of easily comparable pipelines would make composite data analysis easier. Currently, multiple state and local public health laboratories in the USA are adopting MycoSNP (https://github.com/CDCgov/mycosnp-nf) [48], a GATK-based pipeline that uses Nextflow, for conducting genomic epidemiology of *C. auris* infections. Another option for easier composite data analysis is establishing a set of easily comparable pipelines, such as those implemented on Terra. For example, in the human genomics community, a set of functional equivalent pipelines were established producing comparable results, which helped establish a community consensus [49]. In addition, automated analyses of deposited genome sequence data are being developed aimed at routine screening of new data. Examples include the National Center for Biotechnology Information (NCBI) Pathogen Detection tool that now includes *C. auris* [50]. An assembly-based method was included in this study under pipeline 14. Although it was not included in most comparisons, since it generated different output files, the phylogeny generated by this method was comparable with those generated by the reference-based methods. Reassuringly, all methods were able to detect substitutions in the *ERG11* gene linked to resistance.

In designing benchmarking projects for other fungi, we recommend several considerations. First, having high-quality reference genomes is fundamental for any variant calling project. Second, selecting a dataset that is representative of the species population diversity, and also contains some controls such as the same sample or the same sample as the reference genome, as we included here. Third, there needs to be some idea of ground truth, or the true set of SNP variants, which can be built by combining high-quality data sources and evaluating it using control samples. We acknowledge that compared to *C. auris*, most other fungi have more complex genomes, with most having larger genome sizes and higher repetitive content; diploid and heterozygous genomes also add challenges for variant calling. Yet, the relative simplicity of *C. auris* genome, the abundance of genomic data for this pathogen, and the urgent public health need for tools for monitoring the transmission of this fungus make it a good model system for evaluating performances of different variant calling methods. A similar approach can be applied for other more complex fungal genomes.

The availability of cloud-based computational biology platforms and container technologies made it easy to share pipelines with the required computing environment, so that different groups could have easy access to the same methods to generate reproducible results. In this study, we included an example of converting an on-premises pipeline to a cloud-based pipeline (i.e. the Terra platform). Web tools that are accessible to users of all bioinformatics skills levels and focused on public health delivery (e.g. Pathogenwatch, pipeline 14) provide an easy-to-use alternative for tree building and prediction of antimicrobial resistance mutations. Whether they are on the cloud or on-premises, pipelines need to be clearly documented so that others can reproduce them and compare results. We envision that consensus in SNP discovery would greatly benefit our microbial genomics community in future research.

## Supplementary Data

Supplementary material 1

Supplementary material 2

Supplementary material 3

Supplementary material 4

## References

[1] Tsay S, Welsh RM, Adams EH, Chow NA, Gade L (2017). Notes from the field: ongoing transmission of *Candida auris* in health care facilities - United States, June 2016-May 2017. MMWR Morb Mortal Wkly Rep.

[2] Desjardins CA, Giamberardino C, Sykes SM, Yu C-H, Tenor JL (2017). Population genomics and the evolution of virulence in the fungal pathogen *Cryptococcus neoformans*. Genome Res.

[3] Chow NA, Muñoz JF, Gade L, Berkow EL, Li X (2020). Tracing the evolutionary history and global expansion of *Candida auris* using population genomic analyses. mBio.

[4] Ropars J, Maufrais C, Diogo D, Marcet-Houben M, Perin A (2018). Gene flow contributes to diversification of the major fungal pathogen *Candida albicans*. Nat Commun.

[5] Lockhart SR, Etienne KA, Vallabhaneni S, Farooqi J, Chowdhary A (2017). Simultaneous emergence of multidrug-resistant *Candida auris* on 3 continents confirmed by whole-genome sequencing and epidemiological analyses. Clin Infect Dis.

[6] O’Hanlon SJ, Rieux A, Farrer RA, Rosa GM, Waldman B (2018). Recent Asian origin of chytrid fungi causing global amphibian declines. Science.

[7] Islam MT, Croll D, Gladieux P, Soanes DM, Persoons A (2016). Emergence of wheat blast in Bangladesh was caused by a South American lineage of *Magnaporthe oryzae*. BMC Biol.

[8] Nielsen R, Paul JS, Albrechtsen A, Song YS (2011). Genotype and SNP calling from next-generation sequencing data. Nat Rev Genet.

[9] Olson ND, Lund SP, Colman RE, Foster JT, Sahl JW (2015). Best practices for evaluating single nucleotide variant calling methods for microbial genomics. Front Genet.

[10] Cuomo CA (2017). Harnessing whole genome sequencing in medical mycology. Curr Fungal Infect Rep.

[11] McKenna A, Hanna M, Banks E, Sivachenko A, Cibulskis K (2010). The genome analysis toolkit: a MapReduce framework for analyzing next-generation DNA sequencing data. Genome Res.

[12] Li H, Handsaker B, Wysoker A, Fennell T, Ruan J (2009). The sequence alignment/map format and SAMtools. Bioinformatics.

[13] Krusche P, Trigg L, Boutros PC, Mason CE, De La Vega FM (2019). Best practices for benchmarking germline small-variant calls in human genomes. Nat Biotechnol.

[14] Van der Auwera GA, Carneiro MO, Hartl C, Poplin R, Del Angel G (2013). From FastQ data to high confidence variant calls: the genome analysis toolkit best practices pipeline. Curr Protoc Bioinform.

[15] Chow NA, Gade L, Tsay SV, Forsberg K, Greenko JA (2018). Multiple introductions and subsequent transmission of multidrug-resistant *Candida auris* in the USA: a molecular epidemiological survey. Lancet Infect Dis.

[16] Eyre DW, Sheppard AE, Madder H, Moir I, Moroney R (2018). A *Candida auris* outbreak and its control in an intensive care setting. N Engl J Med.

[17] Rhodes J, Abdolrasouli A, Farrer RA, Cuomo CA, Aanensen DM (2018). Author correction: genomic epidemiology of the UK outbreak of the emerging human fungal pathogen *Candida auris*. Emerg Microbes Infect.

[18] Muñoz JF, Gade L, Chow NA, Loparev VN, Juieng P (2018). Genomic insights into multidrug-resistance, mating and virulence in *Candida auris* and related emerging species. Nat Commun.

[19] Bolger AM, Lohse M, Usadel B (2014). Trimmomatic: a flexible trimmer for illumina sequence data. Bioinformatics.

[20] Martin M (2011). Cutadapt removes adapter sequences from high-throughput sequencing reads. EMBnet J.

[21] Li H (2013). Aligning
sequence reads, clone sequences and assembly contigs with BWA-MEM. *ArXiv13033997
Q-Bio*. http://arxiv.org/abs/1303.3997.

[22] Poplin R, Ruano-Rubio V, DePristo MA, Fennell TJ, Carneiro MO (2018). Scaling accurate genetic variant discovery to tens of thousands of samples. bioRxiv.

[23] Felsenstein J PHYLIP (phylogeny inference package) version 3.6.3. Available via the web. http://evolution.genetics.washington.edu/phylip.html.

[24] Kumar S, Stecher G, Tamura K (2016). MEGA7: Molecular Evolutionary Genetics Analysis Version 7.0 for bigger datasets. Mol Biol Evol.

[25] Stamatakis A (2014). RAxML version 8: a tool for phylogenetic analysis and post-analysis of large phylogenies. Bioinformatics.

[26] Swofford D PAUP*.

[27] Sahl JW, Lemmer D, Travis J, Schupp JM, Gillece JD (2016). NASP: an accurate, rapid method for the identification of SNPs in WGS datasets that supports flexible input and output formats. Microb Genom.

[28] Kurtz S, Phillippy A, Delcher AL, Smoot M, Shumway M (2004). Versatile and open software for comparing large genomes. Genome Biol.

[29] DePristo MA, Banks E, Poplin R, Garimella KV, Maguire JR (2011). A framework for variation discovery and genotyping using next-generation DNA sequencing data. Nat Genet.

[30] Birger C, Hanna M, Salinas E, Neff J, Saksena G (2017). FireCloud, a scalable cloud-based platform for collaborative genome analysis: strategies for reducing and controlling costs. bioRxiv.

[31] Katoh K, Toh H (2008). Recent developments in the MAFFT multiple sequence alignment program. Brief Bioinform.

[32] Zheng X, Levine D, Shen J, Gogarten SM, Laurie C (2012). A high-performance computing toolset for relatedness and principal component analysis of SNP data. Bioinforma Oxf Engl.

[33] Lunter G, Goodson M (2011). Stampy: a statistical algorithm for sensitive and fast mapping of illumina sequence reads. Genome Res.

[34] Walker BJ, Abeel T, Shea T, Priest M, Abouelliel A (2014). Pilon: an integrated tool for comprehensive microbial variant detection and genome assembly improvement. PLoS One.

[35] Page AJ, De Silva N, Hunt M, Quail MA, Parkhill J (2016). Robust high-throughput prokaryote *de novo* assembly and improvement pipeline for illumina data. Microb Genom.

[36] Bankevich A, Nurk S, Antipov D, Gurevich AA, Dvorkin M (2012). SPAdes: a new genome assembly algorithm and its applications to single-cell sequencing. J Comput Biol.

[37] Boetzer M, Henkel CV, Jansen HJ, Butler D, Pirovano W (2011). Scaffolding pre-assembled contigs using SSPACE. Bioinforma Oxf Engl.

[38] Boetzer M, Pirovano W (2012). Toward almost closed genomes with GapFiller. Genome Biol.

[39] Seemann T (2014). Prokka: rapid prokaryotic genome annotation. Bioinforma Oxf Engl.

[40] Pruitt KD, Tatusova T, Brown GR, Maglott DR (2012). NCBI reference sequences (RefSeq): current status, new features and genome annotation policy. Nucleic Acids Res.

[41] Muñoz JF, Welsh RM, Shea T, Batra D, Gade L (2021). Clade-specific chromosomal rearrangements and loss of subtelomeric adhesins in *Candida auris*. Genetics.

[42] Bravo Ruiz G, Ross ZK, Holmes E, Schelenz S, Gow NAR (2019). Rapid and extensive karyotype diversification in haploid clinical *Candida auris* isolates. Curr Genet.

[43] Sims D, Sudbery I, Ilott NE, Heger A, Ponting CP (2014). Sequencing depth and coverage: key considerations in genomic analyses. Nat Rev Genet.

[44] Poplin R, Chang P-C, Alexander D, Schwartz S, Colthurst T (2018). A universal SNP and small-indel variant caller using deep neural networks. Nat Biotechnol.

[45] Zook JM, McDaniel J, Olson ND, Wagner J, Parikh H (2019). An open resource for accurately benchmarking small variant and reference calls. Nat Biotechnol.

[46] Welsh RM, Misas E, Forsberg K, Lyman M, Chow NA (2021). *Candida auris* whole-genome sequence benchmark dataset for phylogenomic pipelines. J Fungi Basel Switz.

[47] Walter KS, Colijn C, Cohen T, Mathema B, Liu Q (2020). Genomic variant-identification methods may alter *Mycobacterium tuberculosis* transmission inferences. Microb Genom.

[48] Bagal UR, Phan J, Welsh RM, Misas E, Wagner D (2022). MycoSNP: a portable workflow for performing whole-genome sequencing analysis of *Candida auris*. Methods Mol Biol.

[49] Regier AA, Farjoun Y, Larson DE, Krasheninina O, Kang HM (2018). Functional equivalence of genome sequencing analysis pipelines enables harmonized variant calling across human genetics projects. Nat Commun.

[50] NCBI Resource Coordinators (2017). Database resources of the national center for biotechnology information. Nucleic Acids Res.

