# Supplementary material for: Comparing genomic variant identification protocols for Candida auris
Source: Microb Genom. 2023 Apr 12;9(4):mgen000979. doi: 10.1099/mgen.0.000979 (PMC10210944; doi:10.1099/mgen.0.000979)
Supplement: Supplementary material 1 [file mgen-9-979-s001.pdf]

Supplemental Figures for Li et al, "Comparing genomic variant identification protocols for fungi"

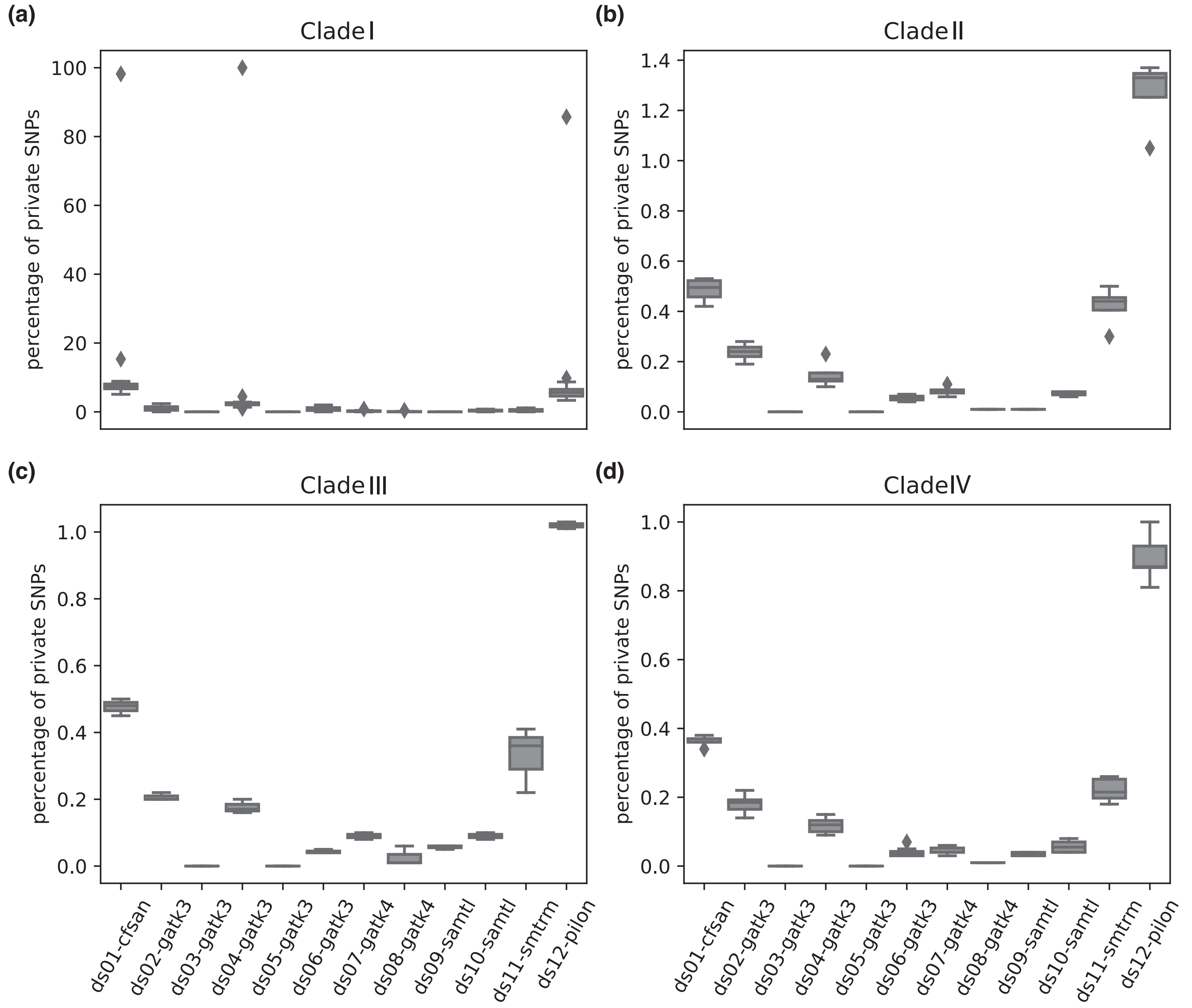

Figure S1. Percentage of private SNPs for each pipeline by clades. Private SNPs were defined as SNPs discovered by only one pipeline. Each panel showed the distributions of percentage of private SNPs for each pipeline for isolate from a specific *C. auris* clade. (**a**) clade I (n=20), (**b**) clade II (n=4), (**c**) clade III (n=3) and (**d**) clade IV (n=8).

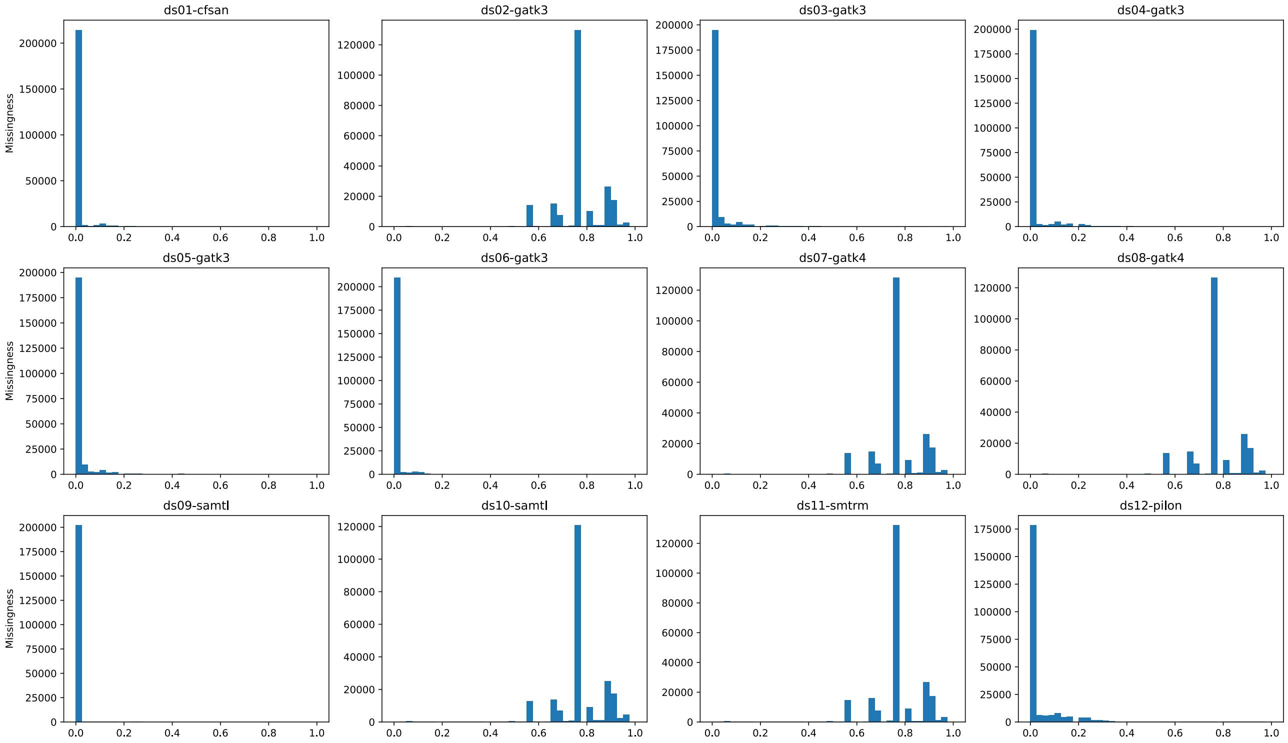

Figure S2. Missingness distribution of each SNP calling pipeline. Horizontal axis shows the missing rate of each site, and the vertical axis indicates number of sites within that frequency bin. Each panel of the plot shows the missingness distribution of a SNP calling pipeline.

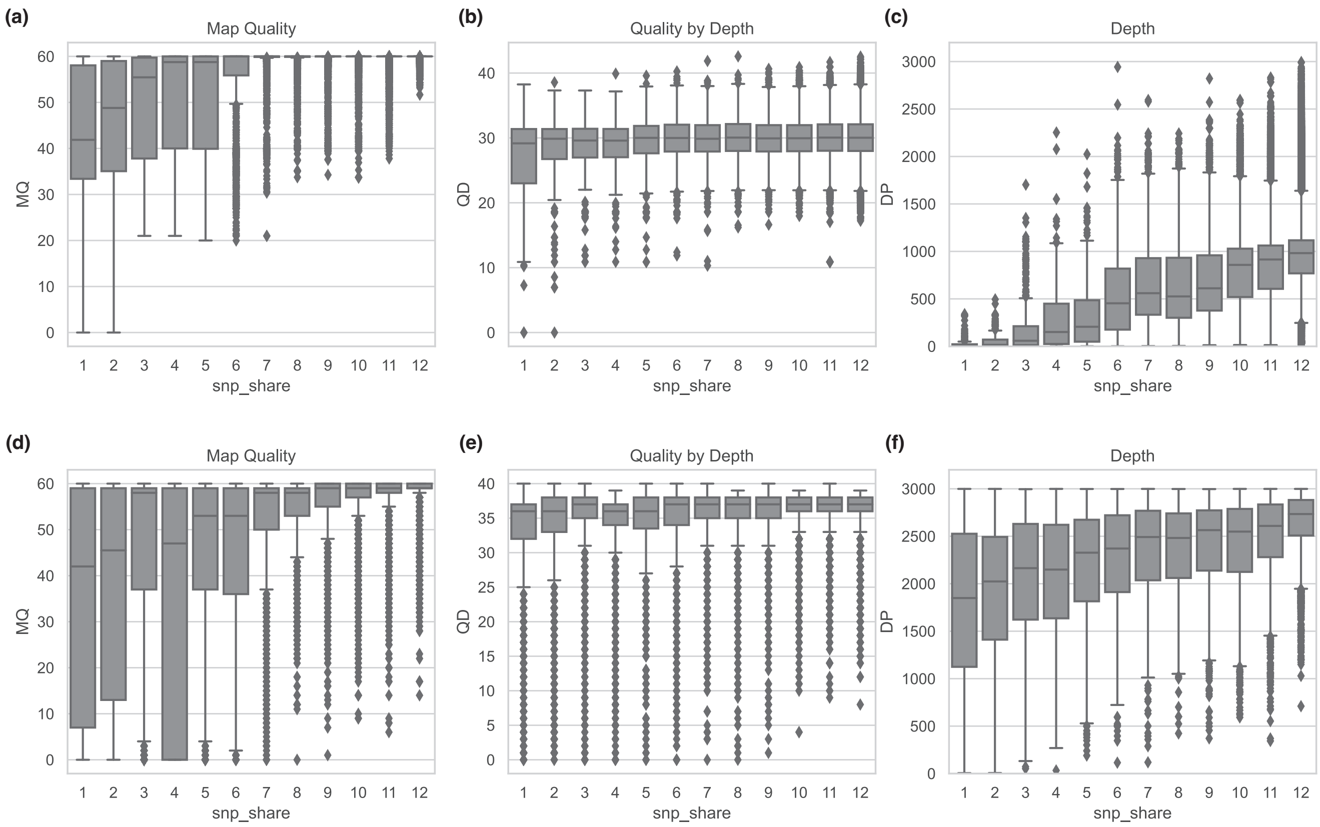

Figure S3. Private SNPs show low mapping quality and read depth. Quality metrics in dataset 7 (**a**, **b** and **c**) and dataset 12 (**d**, **e**, and **f**)**.** Each plot shows the distribution of quality metrics: Map Quality (MQ; **a**, **d**), Quality by Depth (QD; **b**, **e**) and depth (DP; **c**, **f**) across SNPs sharing categories. Horizontal axis shows number of pipelines that discovered each SNP.

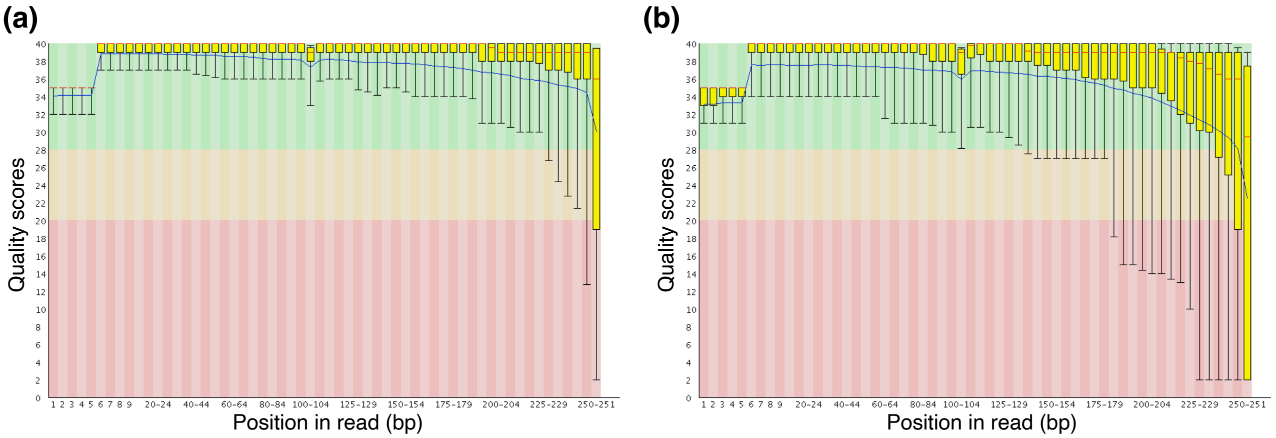

Figure S4. Read quality score distributions. Example (first library of CA01) of read quality score distributions across position of the reads. Panels (**a**) and (**b**) shows the distribution of first and second read in the read pair respectively.
