# Supplementary material for: Comparing genomic variant identification protocols for Candida auris
Source: Microb Genom. 2023 Apr 12;9(4):mgen000979. doi: 10.1099/mgen.0.000979 (PMC10210944; doi:10.1099/mgen.0.000979)
Supplement: Supplementary material 4 [file mgen-9-979-s004.pdf]

````
---
title: "ISHAM WGS pipe matrix analyses"
output: html_notebook
---

```{r}
#import data matrices from local files
data01 =
read.table("~/Documents/CDCProjects/Candida_auris/WGSpipelines/matrices/dataset01")
data02 =
read.table("~/Documents/CDCProjects/Candida_auris/WGSpipelines/matrices/dataset02")
data03 =
read.table("~/Documents/CDCProjects/Candida_auris/WGSpipelines/matrices/dataset03_v3.tx
t")
data04 =
read.table("~/Documents/CDCProjects/Candida_auris/WGSpipelines/matrices/dataset04")
data05 =
read.table("~/Documents/CDCProjects/Candida_auris/WGSpipelines/matrices/dataset05")
data06 =
read.table("~/Documents/CDCProjects/Candida_auris/WGSpipelines/matrices/dataset06")
data07 =
read.table("~/Documents/CDCProjects/Candida_auris/WGSpipelines/matrices/dataset07_v3.tx
t")
data08 =
read.table("~/Documents/CDCProjects/Candida_auris/WGSpipelines/matrices/dataset08")
#data set 9 is paired values; covert to matrix using acast
data09pairs =
read.table("~/Documents/CDCProjects/Candida_auris/WGSpipelines/matrices/dataset09allpair
s")
library(reshape2)
data09 = acast(data09pairs, V1~V2, value.var="V3")#convert the data09 from paired values to a
matrix
data10 =
read.table("~/Documents/CDCProjects/Candida_auris/WGSpipelines/matrices/dataset10_v3.tx
t")
data11 =
read.table("~/Documents/CDCProjects/Candida_auris/WGSpipelines/matrices/dataset11_v3.tx
t")
data12 =
read.table("~/Documents/CDCProjects/Candida_auris/WGSpipelines/matrices/dataset12_v2.tx
t")
data13 =
read.table("~/Documents/CDCProjects/Candida_auris/WGSpipelines/matrices/dataset13_v3.tx
t")
````
````

```
#dataset14 did not merge lanes; use lane1 calls
data14 =
read.table("~/Documents/CDCProjects/Candida_auris/WGSpipelines/matrices/dataset14.txt")

#Assign samples to clades
clade1 <- c("CA03", "CA04", "CA07", "CA08", "CA09", "CA13", "CA14", "CA17", "CA18", "CA20",
"CA21", "CA27", "CA28", "CA29", "CA30", "CA32", "CA34", "CA35") #South Asia
clade2 <- c("CA01", "CA10", "CA11", "CA22") #East Asia
clade3 <- c("CA05", "CA15", "CA23") # Africa
clade4 <- c("CA02", "CA06", "CA12", "CA16", "CA19", "CA24", "CA31", "CA33") # South America

alldata = list(data01, data02, data03, data04, data05, data06, data07, data08, data09, data10,
data11, data12, data13, data14)
datalabels = c("01", "02", "03", "04", "05", "06", "07", "08", "09", "10", "11", "12", "13", "14")

#compare control pairs and make barplots
controls <- matrix(NA, nrow = 14, ncol = 3)
#same patient
for(i in 1:14){controls[i,2]<-alldata[[i]]["CA17","CA18"]}
#same isolate
for(i in 1:14){controls[i,1]<-alldata[[i]]["CA25","CA26"]}
#same outbreak
for(i in 1:14){controls[i,3]<-alldata[[i]]["CA08","CA27"]}

controlsdf = data.frame(controls)
controlsdf$DATASET = datalabels
colnames(controlsdf) <- c("CA25-CA26", "CA17-CA18", "CA08-CA27", "DATASET")

controlmelted <- melt(controlsdf, id.vars="DATASET")
colnames(controlmelted) <- c("DATASET", "COMPARISON", "SNPs")

library(ggplot2)
library(gridExtra)
controlplot1<-ggplot(controlmelted, aes(x=DATASET, y=SNPs, fill=COMPARISON)) +
geom_bar(stat = "identity", position="dodge") + scale_fill_brewer(palette="Set2")

```

```{r}
#subset data to exclude the 3 outliers (datasets 7,8, and 13)
datasubset1 = list(data01, data02, data03, data04, data05, data06, data09, data10, data11,
data12, data14)
datalabels1 = c("01", "02", "03", "04", "05", "06", "09", "10", "11", "12", "14")
```

```
controlsub <- matrix(data=NA, nrow = 11, ncol = 3)
for(i in 1:11){controlsub[i,2]<-datasubset1[[i]]["CA17","CA18"]}
for(i in 1:11){controlsub[i,1]<-datasubset1[[i]]["CA25","CA26"]}
for(i in 1:11){controlsub[i,3]<-datasubset1[[i]]["CA08","CA27"]}
controlsubdf = data.frame(controlsub)
controlsubdf$DATASET = datalabels1
colnames(controlsubdf) <- c("CA25-CA25", "CA17-CA18", "CA08-CA27", "DATASET")

controlsubmelted <- melt(controlsubdf, id.vars="DATASET")
colnames(controlsubmelted) <- c("DATASET", "COMPARISON", "SNPs")

controlplot2<-ggplot(controlsubmelted, aes(x=DATASET, y=SNPs, fill=COMPARISON)) +
geom_bar(stat = "identity", position="dodge") + scale_fill_brewer(palette="Set2")


#output two pdf plots, for all samples (Fig 6a) and excluding 3 outliers (Fig 6b)
theme_set(theme_grey(base_size=6))
pdf("fig6a.pdf", width=4, height=3)
print(controlplot1)

pdf("fig6b.pdf", width=3.14, height=3)
print(controlplot2)
dev.off()
```
